# Identification of a novel lipoic acid biosynthesis pathway reveals the complex evolution of lipoate assembly in prokaryotes

**DOI:** 10.1371/journal.pbio.3002177

**Published:** 2023-06-27

**Authors:** Tomohisa Sebastian Tanabe, Martina Grosser, Lea Hahn, Carolin Kümpel, Hanna Hartenfels, Evelyn Vtulkin, Wanda Flegler, Christiane Dahl

**Affiliations:** Institut für Mikrobiologie & Biotechnologie, Rheinische Friedrich-Wilhelms-Universität Bonn, Bonn, Germany; University of Oxford, UNITED KINGDOM

## Abstract

Lipoic acid is an essential biomolecule found in all domains of life and is involved in central carbon metabolism and dissimilatory sulfur oxidation. The machineries for lipoate assembly in mitochondria and chloroplasts of higher eukaryotes, as well as in the apicoplasts of some protozoa, are all of prokaryotic origin. Here, we provide experimental evidence for a novel lipoate assembly pathway in bacteria based on a sLpl(AB) lipoate:protein ligase, which attaches octanoate or lipoate to apo-proteins, and 2 radical SAM proteins, LipS1 and LipS2, which work together as lipoyl synthase and insert 2 sulfur atoms. Extensive homology searches combined with genomic context analyses allowed us to precisely distinguish between the new and established pathways and map them on the tree of life. This not only revealed a much wider distribution of lipoate biogenesis systems than expected, in particular, the novel sLpl(AB)–LipS1/S2 pathway, and indicated a highly modular nature of the enzymes involved, with unforeseen combinations, but also provided a new framework for the evolution of lipoate assembly. Our results show that dedicated machineries for both de novo lipoate biogenesis and scavenging from the environment were implemented early in evolution and that their distribution in the 2 prokaryotic domains was shaped by a complex network of horizontal gene transfers, acquisition of additional genes, fusions, and losses. Our large-scale phylogenetic analyses identify the bipartite archaeal LplAB ligase as the ancestor of the bacterial sLpl(AB) proteins, which were obtained by horizontal gene transfer. LipS1/S2 have a more complex evolutionary history with multiple of such events but probably also originated in the domain archaea.

## Abstract 2

Lipoic acid is an essential biomolecule found in all domains of life and involved in both central carbon metabolism and dissimilatory sulfur oxidation. This study reveals a novel lipoate assembly pathway in bacteria; integrating this into phylogenomic analyses provides a general picture of the evolution of lipoate biogenesis machineries.

## Introduction

α-Lipoic acid is a cofactor found in all domains of life and is involved in key reactions of central carbon metabolism and dissimilatory sulfur oxidation [1–4]. In this eight-carbon saturated fatty acid, sulfur atoms replace the hydrogen atoms of carbons 6 and 8 of the acyl chain [5]. Only a few, but particularly important, lipoic acid-dependent enzyme systems have been described [2,3,6]. These include 3 α-ketoacid dehydrogenases, such as pyruvate dehydrogenase, whose E2 subunits bind lipoic acid. In the glycine cleavage complex, lipoate is bound to the glycine cleavage H protein (GcvH) [3]. Lipoylated proteins also play an important role in combatting reactive oxygen species [7,8]. In 2018, we discovered another lipoate-binding protein (LbpA) homologous to GcvH (S1 Fig) and demonstrated that it is an essential component of the sulfur-oxidizing heterodisulfide reductase-like (sHdr) system present in a wide range of bacterial and archaeal dissimilatory sulfur oxidizers [2,9].

**Fig 1:**
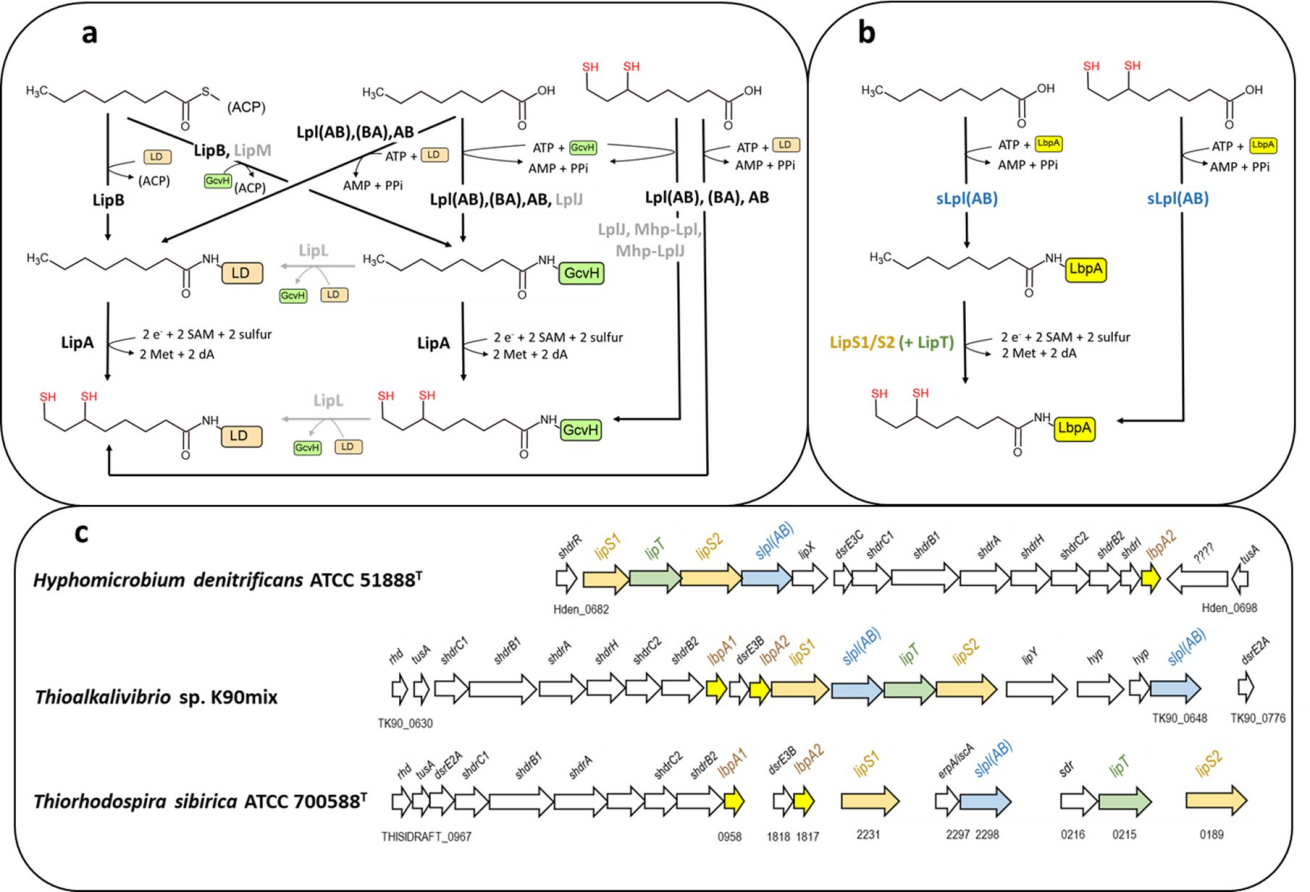
Processes and components of lipoate assembly pathways. (**a**) Main known steps of established lipoate assembly pathways. Enzymes and steps not occurring in *E*. *coli* but described for other organisms are printed in gray. LipM and LipL have been demonstrated in Firmicutes, *B*. *subtilis* [24], *Staphylococcus aureus* [25], and *Listeria monocytogenes* [26], as well as in Tenericutes, *Mycoplasma hyopneumoniae* [15,16]. (**b**) Predicted novel lipoate assembly pathway. The pathway is substantiated by experiments reported here as well as by published work on proteins from the 3 model organisms depicted in **c** [2,9] and by genetic and biochemical work on LipS1 and LipS2 from the archaeon *Thermococcus kodakarensis* [27]. Lipoate:protein ligases from sulfur oxidizers were originally reported not to contain a carboxy-terminal LplB domain based on superposition of the structure modeled for *Thioalkalivibrio* sp. K90mix by using the automated mode of SWISS_Model on *E*. *coli* Lpl(AB). We challenged this view and indeed, modeling by Alphafold [28] as well as sequence alignments yielded clear proof for the presence of the LplB domain (S2 Fig). (**c**) Genetic arrangement of 3 novel systems for lipoate assembly in Proteobacteria. Colors correspond to the biochemical roles as depicted in **b**. For *Ts*. *sibirica* locus tags are given according to JGI-IMG. LipT is an FAD-binding NAD(P)H-dependent oxidoreductase possibly delivering electrons for the LipS1/S2-catalyzed sulfur insertion step. The genes *lipY* and *lipX* encode a putative fatty acid transporter and a putative glutamine amidotransferase, respectively. ACP, acyl carrier protein; GcvH, glycine cleavage system protein H; LbpA, lipoate-binding protein; LD, lipoyl domains of the 2-oxoacid dehydrogenases.

Two posttranslational machineries are known to construct lipoyl moieties [1,3]: The first requires an acyl carrier protein (ACP)-bound octanoyl residue from endogenous fatty acid biosynthesis to be transferred to the ε-amino groups of conserved lysine residues in the accepting apo-proteins. In the second, free lipoate or octanoate are hooked up to the target lysine. Irrespective of the initial step, a sulfur atom must be added to each of the octanoyl C_6_ and C_8_ atoms to complete lipoate biosynthesis (Fig 1A). Using free precursors involves the enzyme lipoate:protein ligase that activates the precursors to lipoyl- or octanoyl-AMP at the expense of ATP before transfer to the target protein. In many bacteria, including *Escherichia coli*, the ligase consists of 2 fused domains, the catalytic domain LplA and the accessory domain LplB [10,11]. We denote these enzymes Lpl(AB) or in the circularly permutated case [12], Lpl(BA). Ligases with tight substrate specificity have been described that transfer free precursors only to GcvH and, in one case, also to the E2 subunit of 2-oxoglutarate dehydrogenase [13–15]. Additional amidotransferases or ligases are then necessary for modification of other lipoyl domains [13,14,16]. Bipartite lipoate:protein ligases forming a functional LplA-LplB heterodimer (denoted LplAB here) have so far been found primarily in archaea [10]. In the absence of free precursors, an octanoyl residue derived from ACP is attached to the target protein by an octanoyltransferase, LipB or LipM [1,16–18]. LipB has been found mainly in Proteobacteria, serves as an all-purpose transferase and provides octanoate or lipoate to all known lipoate-requiring pathways except the sHdr-system [2,19]. LipM has been proposed to occur predominantly in Firmicutes and to transfer octanoyl residues exclusively to GcvH. An amidotransferase, LipL, is required for the transfer of octanoyl or lipoyl moieties from GcvH to the E2-subunits of pyruvate and branched-chain α-ketoacid dehydrogenases [13,14,20]. Despite poor sequence conservation, an evolutionary relationship has been detected between lipoate:protein ligases and octanoyltransferases as well as biotin:protein ligases (BirA) [6]. Once the octanoyl residues arrive at their target proteins, they become substrates for lipoate synthase LipA, a member of the radical S-adenosylmethionine (SAM) superfamily [21], which sequentially adds 2 sulfur atoms in a single reaction, first at position C_6_ and then at C_8_ [1,22,23].

Initial evidence for an alternative pathway for lipoate assembly in prokaryotes emerged, when we showed that the GcvH-like LbpA proteins involved in sHdr-based sulfur oxidation are not modified by the canonical *E*. *coli* and *Bacillus subtilis* lipoyl attachment machineries [2]. Instead, the bacterial and archaeal *shdr-lbpA* clusters are accompanied by a set of genes encoding a specific lipoylation pathway (Fig 1B and 1C) that includes lipoate:protein ligases (sLpl(AB)) and 2 proteins of the radical SAM superfamily, originally termed RadSAM1 and RadSAM2. sLpl(AB) lipoate:protein ligases from sulfur oxidizers not only lipoylate LbpA acceptor proteins from the same organism in vitro but also show cross-species functionality among sulfur oxidizers while failing to recognize lipoyl domains/proteins from organisms lacking components of a sHdr-LbpA sulfur-oxidizing system [2].

Recently, proteins from the thermophilic archaeon *Thermococcus kodakarensis* similar to sLpl(AB) ligase (*Tk-*Lpl-N and *Tk-*Lpl-C) and RadSAM1 and RadSAM2 from sulfur oxidizers (now termed LipS1 and LipS2) were shown to exert octanoate/lipoate:protein ligase and LipA-like lipoate synthase functions, respectively, on chemically synthesized peptide substrates in vitro. Genetic analysis provided further evidence that these proteins are involved in archaeal lipoate biosynthesis [16,27,29] (Fig 1B). We have previously shown that the *slpl(AB)-*encoded protein:lipoate ligase from the gammaproteobacterial sulfur oxidizer *Thioalkalivibrio* sp. K90mix accepts only free precursors, i.e., octanoate or lipoate, and lacks octanoyltransferase activity [2]. The archaeal ligase is also thought to be restricted to free substrates, mainly because ACPs do not occur in archaea [30].

Here, we provide conclusive experimental evidence for the existence of a novel sLpl(AB)-LipS1/S2-based lipoate assembly pathway not only in archaea but also in bacteria and raise the question of whether it is restricted to thermophilic archaea and sulfur-oxidizing bacteria or is of more general importance. To date, no studies have been published on the origin and evolution of lipoate assembly machineries despite the importance of lipoate for almost all living organisms. This prompted us to carry out an exhaustive large-scale analysis including a large fraction of prokaryotic diversity, and in particular, the archaea for which knowledge about lipoate assembly is scarce. We mapped the novel lipoate synthesis pathway on the tree of life revealing an enormously wide distribution. Finally, our analyses show that the novel lipoate synthesis pathway evolved in the archaeal domain.

## Results

### Biochemical and genetic proof for an sLpl(AB)-LipS1/S2-based lipoate assembly pathway in bacteria

The initial proposal of a novel route for maturation of lipoate-binding proteins in bacteria relied on the detection of conspicuous *lipS1-sLpl(AB)-lipT-lipS2* gene clusters, in vitro assays with sLpl(AB) lipoate:protein ligase from a model sulfur oxidizer and genetic complementation studies in *E*. *coli* and *B*. *subtilis* [2]. Here, we set out to collect conclusive experimental evidence for the functionality of the pathway in bacteria. A focus was kept on the biochemically characterized sLpl(AB) ligases from the sulfur oxidizers *Thiorhodospira sibirica* and *Thioalkalivibrio* sp. K90mix [2].

**Fig 2:**
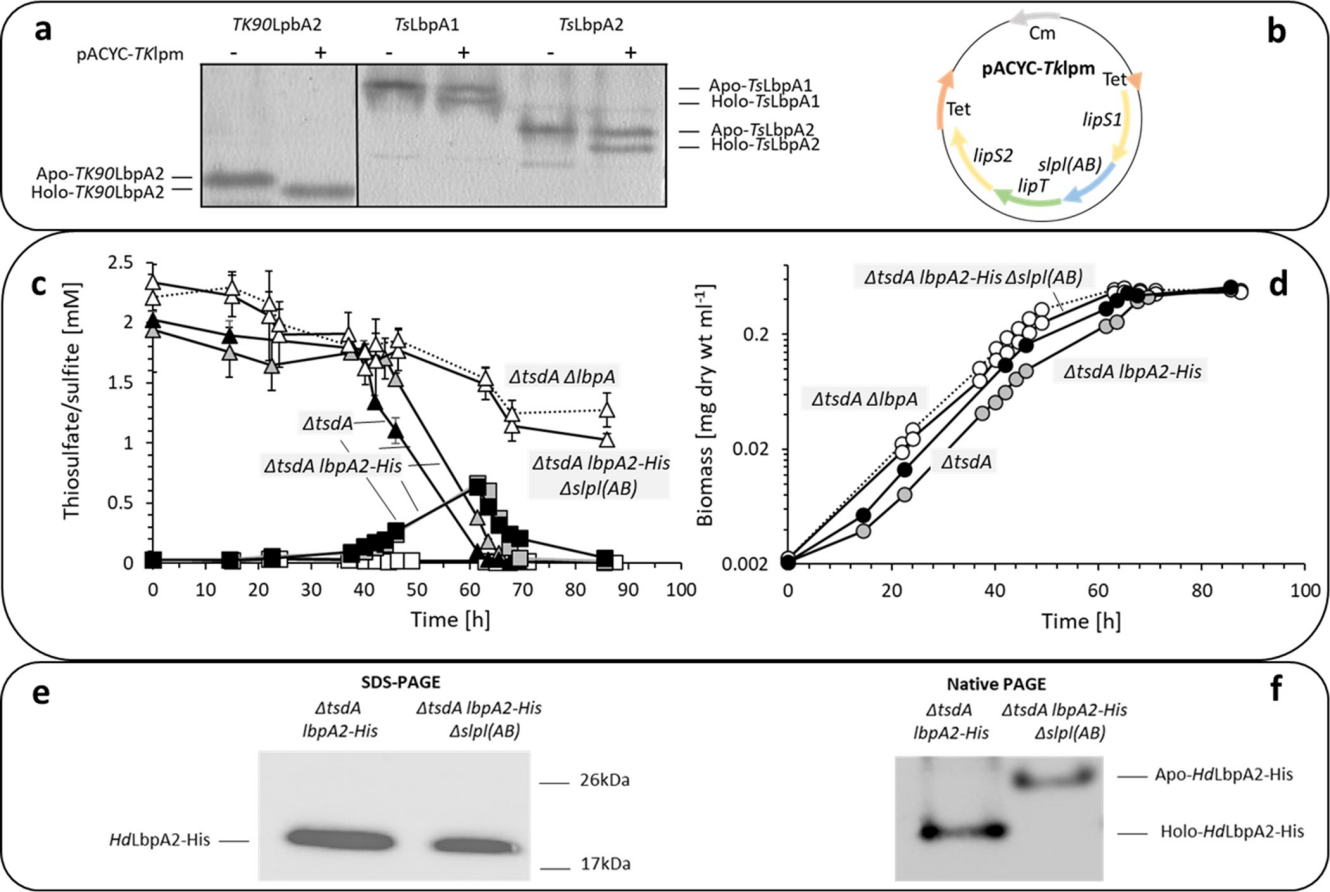
Biochemical and genetic evidence for a novel lipoate assembly pathway in bacteria. (**a**) LbpAs from *Thioalkalivibrio* sp. K90mix and *Ts*. *sibirica* were produced in *E*. *coli* BL21(DE3) Δ*iscR*, a strain designed for improved synthesis of iron-sulfur proteins [32], either with or without a helper plasmid (pACYC-*Tk*lpm) carrying genes *lipS1-slpl(AB)-lipT-lipS2* from *Thioalkalivibrio* sp. K90mix (shown in **b**) under control of the constitutive pACYC184 *tet* promoter. Holo-LbpAs migrate faster in native PAGE due to loss of the positive lysine charge upon modification. In the heterologous host, *Ts*LbpA proteins are—albeit not fully—modified by the assembly proteins stemming from a different species, i.e., *Thioalkalivibrio*. (**c**) Thiosulfate (triangles) and sulfite (boxes) concentrations for 4 different *H*. *denitrificans* strains during growth on methanol (24.4 mM) as a carbon source in the presence of 2 mM thiosulfate. (**d**) Growth of *H*. *denitrificans* strains. Symbols and lines in **c** and **d** correspond to *H*. *denitrificans* strains as follows: filled black symbols, solid lines: *H*. *denitrificans* Δ*tsdA*; symbols filled gray, solid lines: *H*. *denitrificans* Δ*tsdA lbpA2-His;* open symbols, dotted lines: *H*. *denitrificans* Δ*tsdA* Δ*lbpA2*; open symbols, solid lines: *H*. *denitrificans* Δ*tsdA lbpA2-His* Δs*lpl(AB)*. For all measurements, standard deviations based on 3 technical replicates are indicated, but too small to be visible for determination of biomass and sulfite. (**e**) SDS-PAGE of *Hd*LbpA2-His enriched from *H*. *denitrificans ΔtsdA lbpA2-His* (left lane, 2.5 μg protein) and *ΔtsdA Δslpl(AB) lbpA2-His* (right lane, 1.5 μg protein). (**f**) Native gel mobility shift assay for *Hd*LbpA2-His enriched from *H*. *denitrificans ΔtsdA lbpA2-His* (left lane, 2.5 μg protein) and *ΔtsdA Δslpl(AB) lbpA2-His* (right lane, 1.3 μg protein). *Hd*LbpA2-His proteins were visualized after Western blotting using an Anti-His peroxidase conjugate. The data underlying panels **c** and **d** is provided as S1_data.xlsx.

First, 3 Strep-tagged LbpA lipoate acceptor proteins from these 2 bacteria were recombinantly produced in *E*. *coli*, with or without a helper plasmid from which the *Thioalkalivibrio* assembly genes *lipS1-slpl(AB)-lipT-lipS2* were expressed under control of the pACYC184 *tet* promoter (Fig 2A and 2B). Native gel electrophoresis showed the faster mobility expected for holo-LbpAs only when produced in the presence of the helper plasmid. This is due to the lack of the positive charge when the lipoate-binding lysine is modified by covalent attachment of lipoate or octanoate (Fig 2A). Mass spectrometric analyses confirmed posttranslational modification of all 3 LbpA acceptor proteins by a 157-Da mercaptooctanyol moiety in the presence of the helper plasmid (S3 Fig), fully consistent with in vitro results for the archaeal system where LipS2 first catalyzes sulfur attachment at C_8_ of an artificial octanoyllysyl peptide substrate and LipS1 then inserts the second sulfur at C_6_ [29]. Although this last step was not efficiently catalyzed in the heterologous environment, our experiments clearly confirm specific modification of sulfur oxidizer LbpA acceptor proteins by lipoate assembly proteins from a sulfur oxidizer.

In a second approach, 4 strains of the Alphaproteobacterium *Hyphomicrobium denitrificans* were studied. The organism is accessible for manipulative genetics, the necessity of its LbpA2 protein for the oxidation of thiosulfate is documented [2,9] and the respective genes are located in immediate vicinity of a *lipS1-lipT-lipS2-slpl(AB)* cluster (Fig 1C). *H*. *denitrificans* Δ*tsdA* served as the reference strain. It lacks thiosulfate dehydrogenase (TsdA) that catalyzes the formation of the dead-end product tetrathionate. Thus, the strain can oxidize thiosulfate exclusively via the sHdr-LbpA pathway [9,31] that substantially simplifies its elucidation by reverse genetics. When grown in the presence of methanol as a carbon source and thiosulfate as an additional electron source, it excretes toxic sulfite, which causes growth retardation [31]. Functionality of the sHdr-LbpA pathway is thus easily detectable by sulfite formation and diminished growth rate (Fig 2C and 2D). The second strain studied carries a Δ*lbpA2* deletion in a Δ*tsdA* background, is unable to oxidize thiosulfate, and served as control. In the third strain, *lbpA2-His*, encoding carboxy-terminally His-tagged LbpA2, replaces the original *lbpA* gene in the chromosome of *H*. *denitrificans* Δ*tsdA*, so that LbpA2 can be purified from this strain. The fourth strain carries an *in frame* deletion of *slpl(AB)* in a Δ*tsdA lbpA2-His* background. Thus, the modification of the lipoate acceptor LbpA2 can be compared in presence or absence of the sLpl(AB) ligase. *H*. *denitrificans ΔtsdA* and *ΔtsdA lbpA2-His* oxidized thiosulfate completely and excreted up to 0.6 mM sulfite (Fig 2C and 2D). This demonstrates that the addition of the carboxy-terminal His-tag does not prevent proper function of the LbpA2 protein. In contrast, in both, the *ΔtsdA ΔlbpA2* and the *ΔtsdA lbpA2-His Δslpl(AB)* strains, thiosulfate degradation was very slow, sulfite formation was not observed (Fig 2C), and the growth rates were higher than those for strains *ΔtsdA* and *ΔtsdA lbpA2-His* (Fig 2D). This confirmed the crucial function of LbpA2 in the cytoplasmic sHdr-LbpA sulfur oxidation pathway and more importantly, showed that the absence of sLpl(AB) lipoate:protein ligase had the same effect as the complete absence of the LbpA2 protein, strongly suggesting that sLpl(AB) ligase is essential for the modification and thus the proper function of LbpA2. We found evidence in support of this hypothesis by enriching the LbpA2 acceptor proteins from the *H*. *denitrificans* strains *ΔtsdA lbpA2-His* and *ΔtsdA lbpA2-His Δslpl(AB)* producing them with a His-tag and comparing their behavior in SDS and native PAGE. While the *H*. *denitrificans* strain lacking sLpl(AB) ligase produced only apo-LbpA2, the holo-protein was produced in the strain containing the complete assembly pathway as evident from the native gel mobility shift (Fig 2E and 2F).

### Lipoate assembly systems are unevenly distributed in bacteria and archaea

The experiments described above clearly established the functionality of a LipS1/S2-based lipoate assembly pathway in bacteria. We now asked how lipoate:protein ligases and octanoyltransferases (LipB, LipM) and the different lipoate synthases (LipA, LipS1/S2) are distributed among the prokaryotes and analyzed the genomes available in the Genome Taxonomy Database (GTDB, release R207). In GTDB, all genomes are sorted according to validly published taxonomies. In order to accurately identify and discriminate the enzymes involved in lipoate assembly pathways, a task severely hampered by the fact that central components are part of very large multi-protein families, we used HMS-S-S, a tool that specifically finds sulfur metabolism-related proteins [33] and extended it with publicly available HMMs for canonical lipoate synthesis enzymes as well as for well-studied lipoate-binding proteins, such as GcvH (S1 Table).

**Fig 3:**
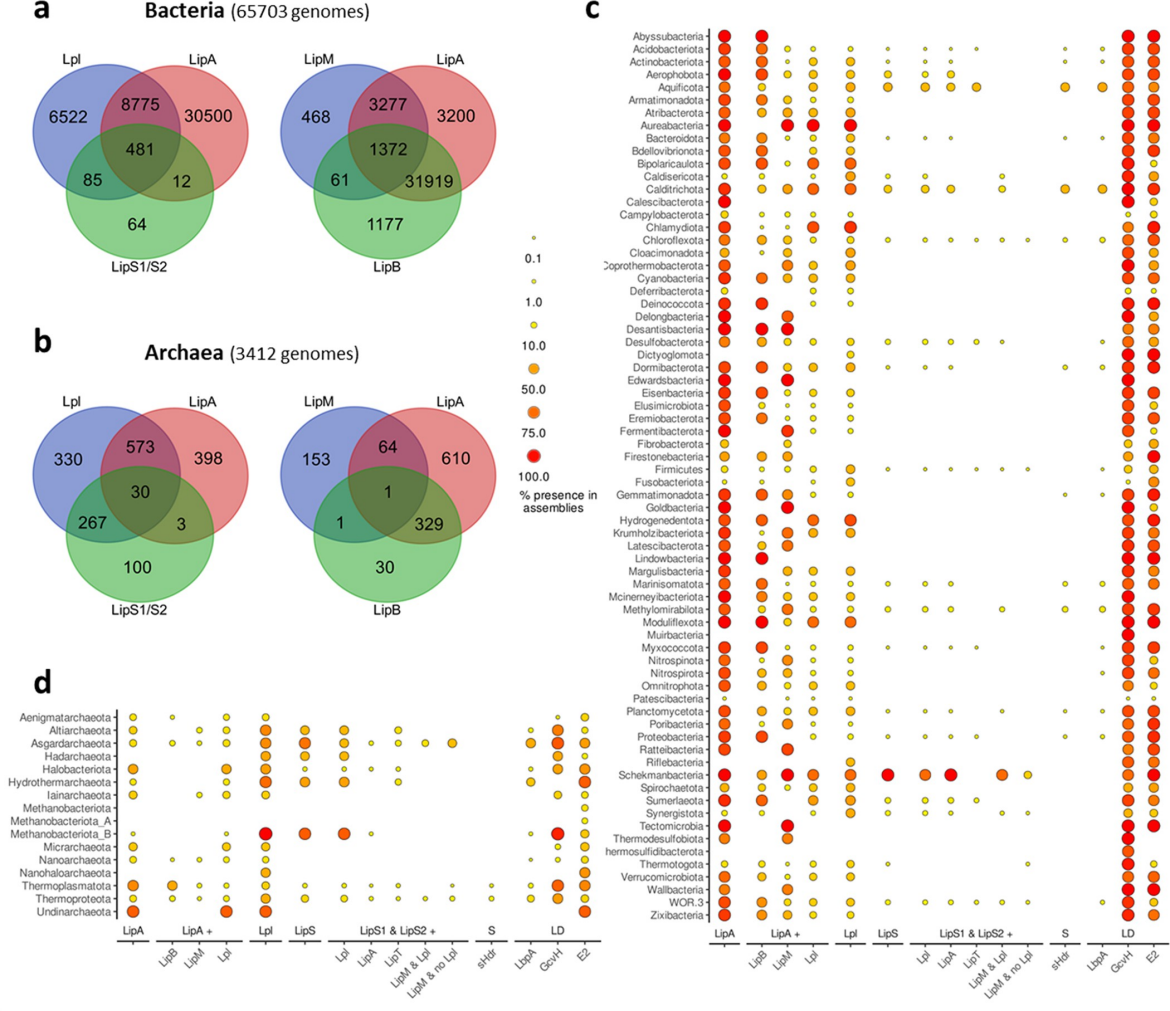
Taxonomic distribution of the lipoate synthesis systems, lipoate scavenging, and lipoate requiring proteins. Venn diagrams show the abundance and overlap of lipoate:protein ligases (Lpl), octanoyl transferase (LipB, LipM), and lipoate synthases (LipA, LipS1/S2) in the bacteria **(a)** and the archaea **(b).** Panels **c** and **d** visualize the taxonomic distribution of these enzymes, the sulfur-oxidizing sHdr system (S) and lipoate-binding domains (LD). For each bacterial **(c)** and archaeal phylum **(d)**, the percentage of genomes possessing these proteins is indicated by dots of different sizes and colors. Note that the proportion was normalized to the size of the phylum and does not show absolute counts or overall phylum size. The data underlying parts **a** and **b** are provided in S2 and S3 Tables, respectively. S4 Table supplies the data underlying parts **c** and **d**.

In both prokaryotic domains, scavenging of free lipoate or octanoate by lipoate:protein ligase is widespread (Fig 3). Lpl/LipA combinations were found in only 14% and 8% of the bacterial and archaeal genomes, respectively, while Lpl/LipS1/S2 were found in 0.9% and 17%, respectively, leaving the majority of prokaryotes unable to use free octanoate for lipoate assembly (Fig 3A and 3B). In bacteria, de novo lipoate synthesis by LipB/LipA or LipM/LipA is more common than lipoate scavenging (Fig 3). The co-occurrence of both octanoyltransferases, LipB and LipM, is very rare and there is usually no strict correlation between phylum and enzyme type. Contrary to previous assumptions [19], LipB is found in the Firmicutes and LipM is not restricted to a specific phylum. The Proteobacteria, together with a few other phyla, are the exception rather than the rule, containing only LipB and never LipM (Fig 3C). As mentioned in the Introduction, *B*. *subtilis* octanoyltransferase LipM is specific for GcvH and an amidotransferase (LipL) is required for modification of other lipoyl-binding domains/proteins. Surprisingly, not all bacteria, including Firmicutes, which carry out LipM/LipA-based lipoate biosynthesis, also possess LipL, although lipoate-binding domains/proteins are encoded in these genomes (S2 and S3 Tables). This suggests a much broader substrate specificity of the octanoyltransferase LipM than previously described or the existence of (an) unknown amidotransferase(s) that functionally replace(s) the amidotransferase LipL. Among the archaea, octanoyltransferase/lipoate synthase combinations LipB/LipA or LipM/LipA were also present (Fig 3), suggesting de novo lipoate synthesis from ACP-bound octanoate. This was unexpected, because ACP is generally absent in archaea [34–36] and cannot serve as direct octanoate donor. Coenzymes A or M are possible alternatives [35–37].

In both prokaryotic domains, the LipS1/S2-type of lipoyl synthase is less common than LipA lipoate synthase but remarkably widespread (Fig 3). Co-occurrence of lipoate synthase LipA is a common feature for *lipS1/S2-*containing bacteria but rare in LipS1/S2-encoding archaea. Our analyses do not confirm the proposal that LipS1/S2 are specific to thermophilic archaea [27]. The Asgardarchaeota, in which LipS1/S2 is more abundant than LipA, are a counter-example, as all sequences, including that of the only cultured representative *Candidatus* Prometheoarchaeum syntrophicum, are from temperate to cold environments [38,39]. Halobacteriota and Altiarchaeota genomes with *lipS1/S2* also stem from temperate/cold habitat samples [40,41]. In addition, LipS1/S2 is present in many mesophilic bacteria, i.e., *H*. *denitrificans*, *Thioalkalivibrio* sp., or *Sporomusa* sp.

### LipS1/S2-type lipoate synthases and their cooperation partners

Regarding the novel lipoate synthesis pathway, our analyses confirm that *lipS1*/*S2* genes are often associated with genes for lipoate:protein ligases (Figs 1C and 3) [2], usually with a Lpl(AB) domain structure (S5 Table). A *lipS2-lplA-lplB-lipS1* arrangement seems typical for Archaea but is also found with some rearrangement in the bacterial phyla Chloroflexota, Aerophobota, and Synergistota. Occasionally, direct linkage of genes for canonical lipoate synthesis with *lipS1/S2* is observed, e.g., in several *Sporomusa* species (phylum Firmicutes). In some LipS1/S2-containing bacteria (e.g., members of the Schekmanbacteria, Synergistota, and Thermotogota) and archaea (members of the Asgardarchaeota, Thermoproteota, and Themoplasmatota), a gene for lipoate:protein ligase is absent (Fig 3 and S2 Table). Instead, *lipS1/S2* co-occur with a gene for octanoyltransferase LipM. In the bacterial cases, this implies that LipS1/S2 insert sulfur into target proteins that have been octanoylated by a transferase reaction. For the archaea, as discussed above, the question of the substrate for the LipM homologs is unresolved.

The *lipT* encoded FAD-containing oxidoreductase is a likely candidate to provide electrons, probably derived from NAD(P)H, for the reductive sulfur insertion catalyzed by LipS1/S2 (Fig 1). Indeed, *lipT* occurs almost exclusively in bacteria containing *lipS1/S2* (91% of the cases), often in a *lipS1-lpl(AB)-lipS2-lipT* arrangement (Fig 1C and S5 Table). The picture is different for archaea, where only 22.3% of the *lipT*-containing genomes also contain *lipS1* and *lipS2* (S4 Table). Approximately 53% and 17% of the LipS1/S2-containing bacteria and archaea, respectively, also encode LipT (S2, S3 and S4 Tables).

The *lipS1/S2* genes were first detected in bacterial and archaeal sulfur oxidizers that use the sHdr pathway for sulfur oxidation [2]. LbpA proteins are essential components of this pathway [2] and are encoded in *shdr*-containing genomes with very few exceptions, probably due to incompleteness of the respective assemblies (Fig 3 and S2 and S3 Tables), raising the question of whether the assembly of LbpA proteins is strictly dependent on LipS1/S2. While the majority of sHdr-containing prokaryotes are indeed equipped with LipS1/S2 (74.3% and 88.6% for bacteria and archaea, respectively; Fig 1C and S2, S3 and S4 Tables), the reverse is not true, i.e., LipS1/S2 are not restricted to sulfur oxidizers (42% and 8% of LipS1/S2-containing bacteria and archaea, respectively, have sHdr; S2, S3 and S4 Tables).

### Evolution of lipoate:protein ligases and octanoyltransferases

**Fig 4:**
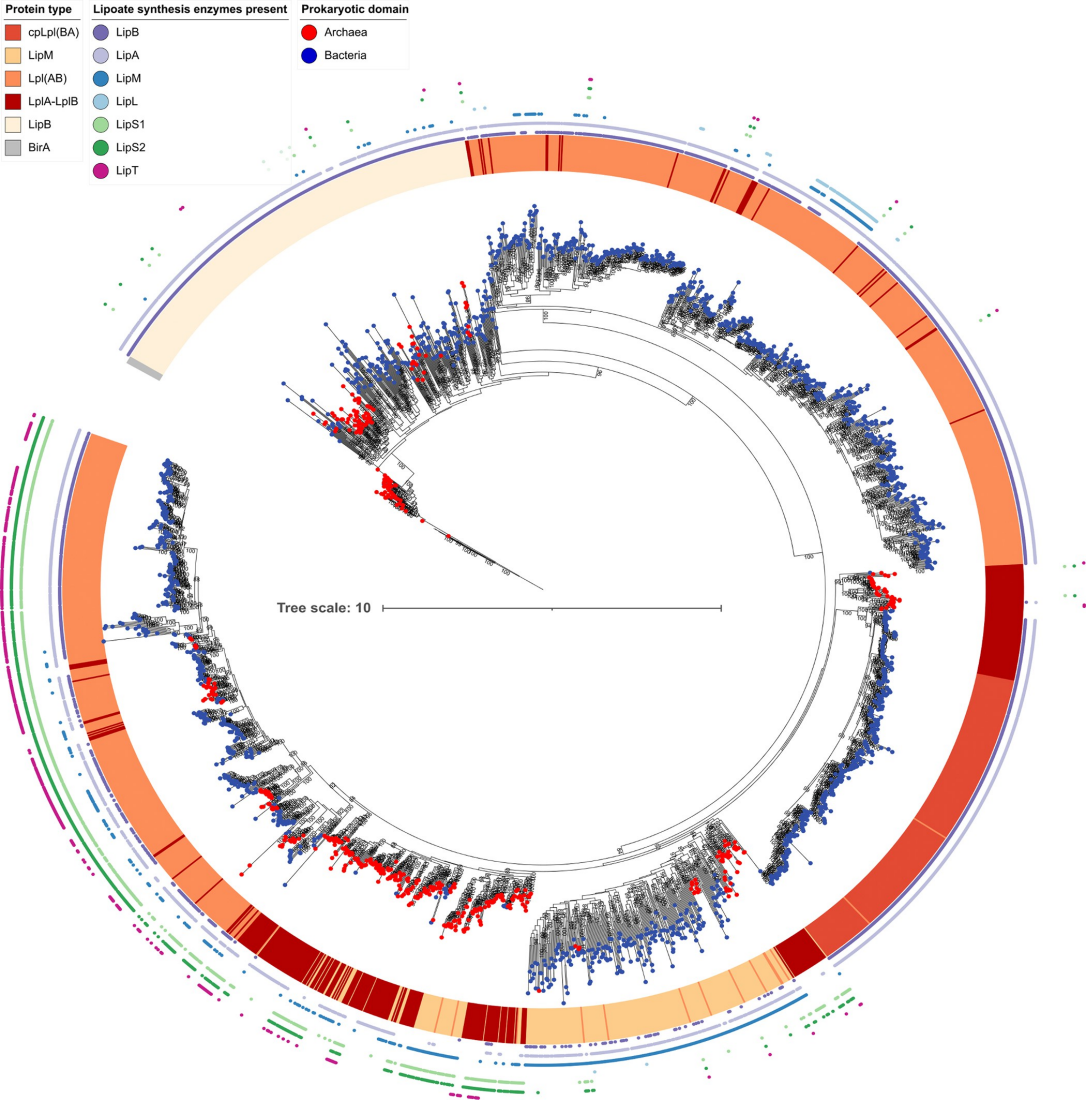
Rooted phylogenetic tree for the complete lipoate:protein ligase/octanoyltransferase dataset. The tree was rooted with the structurally related biotin ligase BirA as an outgroup. Red or blue dots placed on each leaf identify the source organisms as archaea or bacteria, respectively. The ligase/transferase type is color-coded in the next circle. In the outermost rings, the presence of other lipoate synthesis enzymes occurring in the same genome is labeled. The data underlying this figure is provided in Supplementary S3 Data.

All lipoate:protein ligases and octanyoltransferases belong to the cofactor transferase family PF03099. Calculating rooted phylogenetic trees for these proteins was expected to bring new insights into the origin and evolution of these enzymes. If they initially originated in archaea, the tree should be a priori rooted in the archaeal domain and similarly for bacteria. The structurally related biotin ligase BirA, which is also a member of the cofactor transferase family, was chosen as a suitable outgroup to root the tree. The tree for the complete lipoate:protein ligase/octanoyltransferase dataset contains 3 clearly delineated clades with high bootstrap support (Fig 4). The first clade contains the bacterial and archaeal LipB octanoyltransferases and resides between the BirA root and all other analyzed proteins. The second clade harbors a group of lipoate:protein ligases derived exclusively from bacteria with LipA but usually without LipS1/S2 lipoate synthase. Broad substrate range *E*. *coli* Lpl(AB) as well as the narrow substrate range ligases LplJ from *B*. *subtilis*, and Mhp-LplJ and Mhp-Lpl from *M*. *hyopneumoniae* reside in this clade.

For the proteins of clade 3, an archaeal origin is inferred, since bipartite LplABs ligases from the archaeal phyla Thermoproteota and Thermoplasmatota including the characterized *Thermoplasma acidophilum* enzyme [10] are the deepest branching sequences. Three well-supported subgroups (bootstrap ≥92) branch off from these, each again with deep-branching archaeal proteins. In the first subgroup, bipartite LplABs from Nano- and Haloarchaeota form the deepest branches, which are immediately adjacent to LplABs from Burkholderiales (class Gammaproteobacteria according to GTDB). The remaining sequences in the subgroup are circularly permuted Lpl(BA) proteins, nearly exclusively stemming from Actinomycetota and including the characterized *S*. *coelicolor* Lpl(BA) [12]. This topology suggests an evolutionary history with lateral transfer of LplAB from Archaea to Gammaproteobacteria followed or accompanied by rearrangement of the gene order and final fusion of the genes upon transfer to the phylum Actinomycetota, where the gene was then vertically transmitted. The second subgroup consists of archaeal and bacterial bipartite LplAB ligases and LipM-type octanoyltransferases and provides insights into the origin of LipM: (1) Archaeal LplAB ligases, mostly encoded near *lipS1/lipS2* and including the characterized *Thermococcus kodakarensis* protein [30], are the most deeply branching sequences and appear to be the ancestors of a large number of bacterial LipMs, which thus arose from a single interdomain horizontal gene transfer event. A scenario is supported in which archaeal LplAB lost its accessory peptide LplB, developed into LipM and simultaneously or later moved into a bacterial host. (2) In the remaining part of the subgroup, many archaeal and some bacterial LplABs ligases are mixed with many archaeal and some bacterial LipM octanoyltransferases, indicating that the described horizontal gene transfer, loss of LplB and transformation of the remaining catalytic domain LplA into an octanyoltransferase was not a singular event but happened multiple times. The third subgroup of clade 3 is made up of even further archaeal and bacterial LplABs and Lpl(AB) lipoate protein ligases, the vast majority of which originate from organisms containing LipS1/S2. The genetically and biochemically characterized sLpl(AB) ligases from proteobacterial sulfur oxidizers fall into this group.

**Fig 5:**
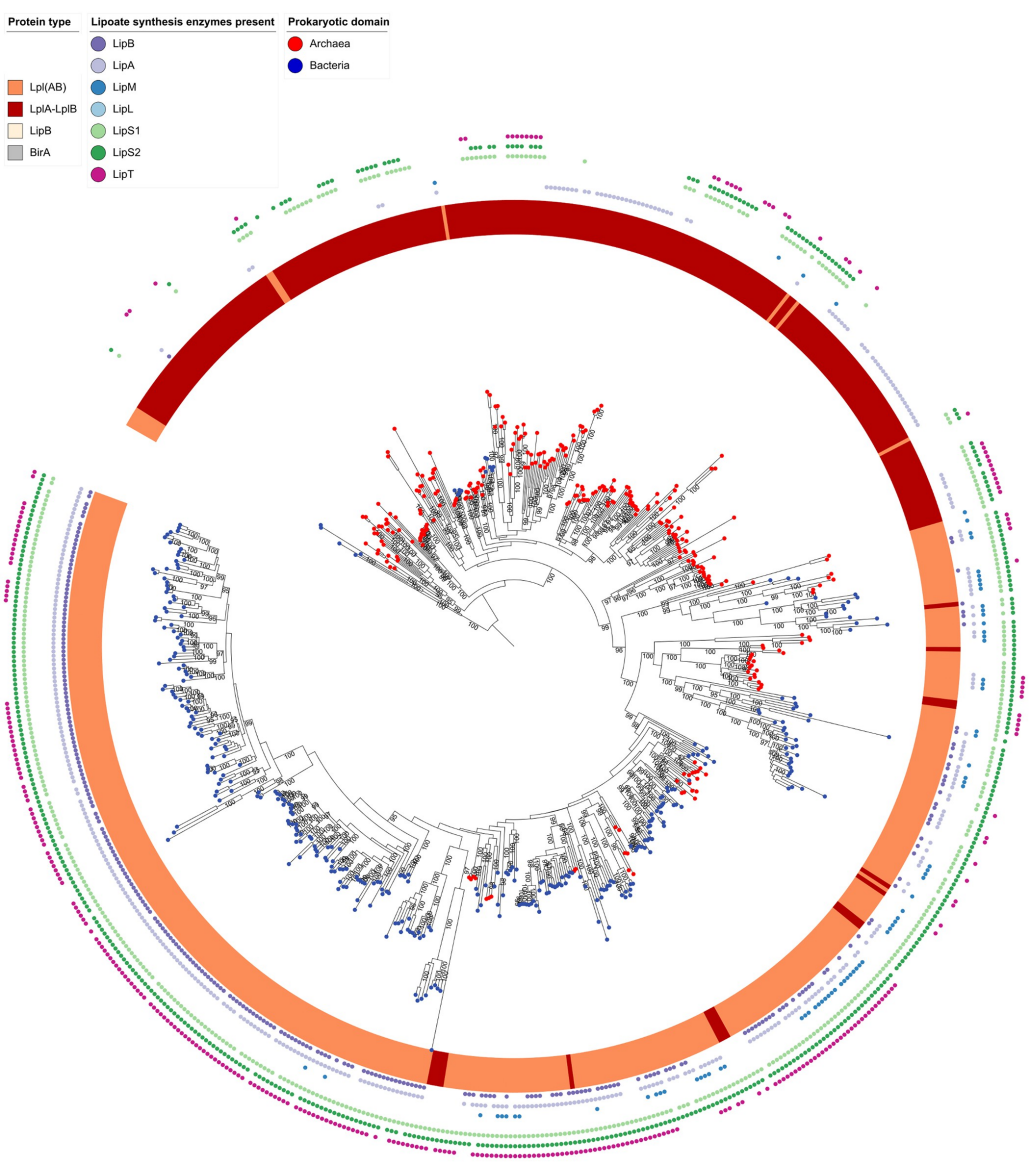
Phylogeny for clade 3 lipoate:protein ligases without LipM and cpLpl(BA). LipMs do not have LplB domains and their sequences are consequently shorter. If a sequence is incomplete, parts of the information used to calculate the phylogenetic tree are missing. This can lead to erroneous estimates of the relationships between sequences and can bias the result and weakens statistical significance of the calculation. In addition, Lpl(BA) clearly shows an individual evolution and may also cause weakening of statistical support. The data underlying this figure is provided in S3 Data.

We challenged the idea that clade 3 has an origin within the archaea by calculating 3 separate trees for this group. The trees were rooted by the most closely related, similarly sized and biochemically characterized bacterial lipoate:protein ligases from clade 2 and confidence levels were increased by not including LipM octanoyltransferases and/or circular permuted cpLplBA lipoate:protein ligases (Figs 5, S4 and S5). The trees have a high to very high bootstrap support especially for the higher order splits and all 3 indeed show a root in the archaeal domain with Thermoproteota and Thermoplasmatota proteins as the deepest branching sequences. Moreover, it is clear that bacterial Lpl(AB)s, which co-occur in the same organism with LipS1/S2, originate from an archaeal ancestor. Several horizontal gene transfer events are also evident. The 2 earliest were transfer of LplAB ligases from Hadarchaeota to Synergistota and from Altiarchaeota to Chloroflexota. On the other hand, several transfers from bacteria back to archaea can be delineated, e.g., into members of the Thorarchaeota, Baldrarchaeota, Jordarchaeota, Sifarchaeota, Thermoplasmatota, and Thermoproteota.

### Phylogenetic analysis of the radical SAM proteins LipS1 and LipS2

**Fig 6:**
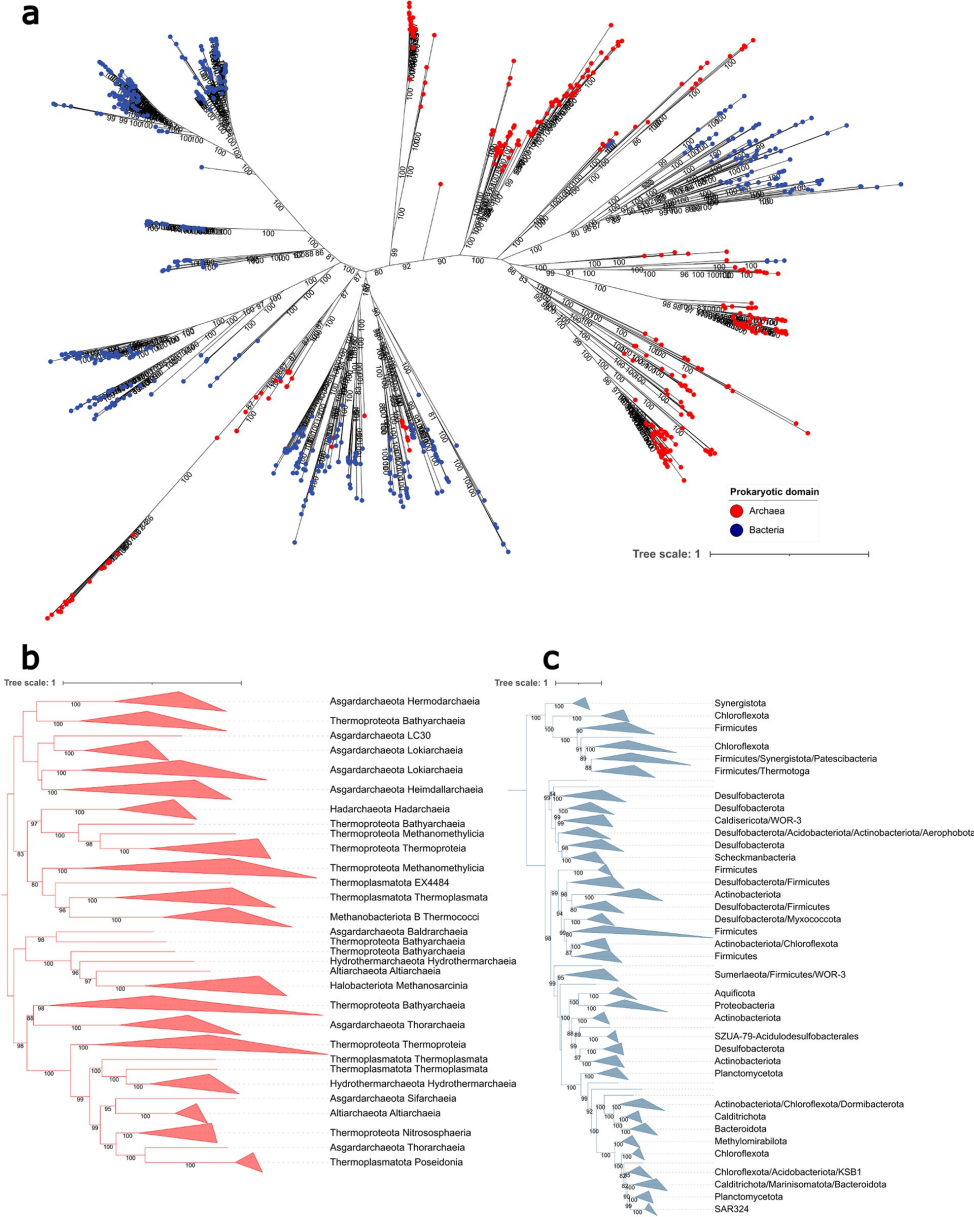
Phylogenetic trees for LipS1/S2. (**a**) To investigate the evolution of LipS1 and LipS2, their sequences were concatenated, as both units are usually found in synteny, are catalytically active together and should therefore be under the same evolutionary pressure [29]. Incomplete sequences and concatenated sequences from genomes lacking either LipS1 or LipS2 were removed from the analysis. The lower panels show schematic representation of phylogenetic trees generated using only archaeal sequences (red, **b**) or bacterial sequences (blue, **c**). Bacterial clades represented by single sequences were left out to increase readability. The data underlying this figure is provided in S3 Data.

The tree obtained for the concatenated LipS1/S2 proteins neither shows a 2 domain split nor a long branching separation, as would be expected for an ancient protein that already existed in a universal common ancestor [42]. This indicates an origin of LipS1/S2 either in the bacterial or in the archaeal domain, followed by horizontal gene transfer into the other domain [43,44]. To obtain further insights, separate trees were constructed for the bacterial and the archaeal proteins (Fig 6B and 6C). If LipS1/S2 lipoate synthase originated in either the archaea or the bacteria and was inherited vertically, the domain-specific tree should essentially follow the taxonomy of that domain. However, neither the tree for archaeal LipS1/S2 nor that for the bacterial equivalents fit this concept. With the exception of Proteobacteria and Aquificota, monophyletic clusters were not recovered within the bacteria (Fig 6B) and among the archaea only the LipS1/S2 lipoyl synthases from the Hadarchaeota, Methanobacteriota, and Halobacteriota appear monophyletic (Fig 6C). The trees indicate that LipS1/S2 has undergone multiple gene transfers between and within the 2 prokaryotic domains.

Finally, some insight into the origin of LipS1/S2 lipoate synthase was gained by rooting separate LipS1 and LipS2 trees by the radical SAM enzyme biotin synthase BioB, which belongs to the same megacluster of similarity and, just like each of the LipS proteins, catalyzes the insertion of a single sulfur atom [29,45]. For LipS2, the resulting tree topology was rather well supported and placement of BioB in the split between 2 archaeal clades was stable (S6 Fig). This points at an origin of LipS2 in the archaea. The final topology of the rooted LipS1 tree is consistent with an archaeal origin but bootstrap support is not high enough to draw firm conclusions (S6 Fig).

## Discussion

Our work proves the existence of a LipS1/S2-based lipoylation pathway not only in archaea but also in bacteria. In both prokaryotic domains, the pathway starts with the incorporation of a free octanoic acid and then LipS1/S2 insert 2 sulfur atoms. The lipoate:protein ligase that catalyzes the first step originates from archaea and thus has a completely different evolutionary history than the previously known Lpl(AB) enzymes in clade 2, which are exclusively found in bacteria. For LipS1/S2, which catalyze the second step, an archaeal origin is also likely. The majority of bacteria that use reduced sulfur compounds as electron donors by the sulfur-oxidizing heterodisulfide reductase-like (sHdr) complex in conjunction with LbpA proteins, are equipped with the originally archaeal lipoate synthesis machinery. Since heterodisulfide reductases are enzymes typical of methanogenic archaea, co-transfer of the genes seems a likely possibility.

Our large-scale integrated phylogenomic analyses open new perspectives on the general evolution and the complexity of lipoate synthesis. Not only do they reveal a much wider distribution of lipoate assembly systems than expected, in particular, the novel sLpl(AB)–LipS1/S2 pathway, and indicate a highly modular nature of the enzymes involved, with unforeseen combinations, but they also provide a new framework for the origin and subsequent evolution of lipoate assembly systems in the 2 prokaryotic domains. Our results are relevant to the entire diversity of life, since the machineries for lipoate synthesis in the mitochondria and plastids of eukaryotes are all of prokaryotic origin and may bear more complexity than currently assumed [1,46,47]. Contrary to the intuitive assumption, mitochondrial LipAs are more closely related to the LipA lipoate synthases of archaea than to those of α-proteobacteria [47]. In addition, plastidial LipAs are not most closely related to those of cyanobacteria, as would be expected if they were derived from the primary endosymbiont that led to the first plastid. Instead, they form a sister group to the mitochondrial LipAs and probably arose by a gene duplication [47]. Furthermore, protozoa with mitochondria and plastids require 2 different ligases for lipoate metabolism in the respective organelles [1].

The tripartite topology of the phylogenetic tree for the octanyltransferases and lipoate:protein ligases has provided particularly important insights. LipB octanoyltransferases (clade 1) are close to the root of the tree and thus represent the ancestors of all the other enzymes. Clade 1 gave rise to 2 other groups, one of which evolved in bacteria (clade 2) and the other is probably of archaeal origin (clade 3). In clade 2, the acquisition of an additional gene encoding LplB, and in many cases its fusion with the original LipB octanoyltransferase unit, allowed the conversion of an octanoyltransferase into an ATP-dependent lipoate:protein ligase. The most deeply branching proteins in clade 3 also have additional LplB, suggesting that the acquisition of the corresponding gene and functional transformation into a ligase was again the starting point for further evolution. Our conclusions are consistent with previous suggestions, based on structural data, that the lipoate:protein ligase LplA initially lacked an LplB unit, which was first acquired as a separate unit before fusion occurred [48].

We infer that bipartite LplAB enzymes represent the most original lipoate:protein ligase in archaea. Free lipoate may therefore have been the primary source of protein lipoylation in ancient archaea. There was no lipoyl synthase in these organisms, as we still find today, for example, in *Thermoplasma acidophilum*. Over time, lipoyl synthases appeared in archaea, either LipA or LipS1/S2 and very rarely both, enabling the use of octanoate. Gene clusters then formed, as we find in almost all recent archaea. In bacteria, clade 2 Lpl(AB) ligases appear to co-operate with LipA and in those bacteria that encode sLpl(AB) ligase, the genes for LipS1/S2 are almost always also present. Genetic linkage of the genes in operons is the usual case. These observations, together with the highly probable archaeal origin for sLpl(AB) ligase and LipS1/S2, suggest that the genes were transferred together from archaea to bacteria, possibly even together with genes for their specific lipoylation substrates. Such a scenario also fully explains why the canonical Lipl(AB)/LipA combination and the sLpl(AB)/LipS1/S2 system can coexist in the same organism and specifically lipoylate only their cognate substrates. Further experimental work on bacterial and also archaeal lipoate:protein ligases and their potential substrates will allow testing these functional hypotheses.

Lipoate-requiring reductive glycine synthesis was placed at the basis of the tree of life, as part of the phenotype of the last common ancestor [19]. Accordingly, the last common ancestor has been proposed to contain radical SAM-dependent enzymes and LipA lipoyl synthase is considered among the most ancient enzymes in this class [21]. In contrast, genes encoding LipS1/S2 are absent in most phyla close to the root of the archaeal domain [49] (Iainarchaeota, Nanohaloarchaeota, Nanoarchaeota, Aenigmatarchaeota, and Micrarchaeota) and likely emerged later than LipA.

In conclusion, our results pave the way for further work on lipoate biochemistry as a key process for ancient and modern life.

## Methods

### Bacterial strains, plasmids, primers, and growth conditions

S6 Table lists the bacterial strains and plasmids that were used for this study. *E*. *coli* strains were grown on complex lysogeny broth (LB) medium [50] or on glycerol-containing M63 minimal medium [51] under aerobic conditions at 37°C unless otherwise indicated. *E*. *coli* 10β was used for molecular cloning. *E*. *coli* BL21 (DE3) was used for recombinant protein production. *H*. *denitrificans* strains were cultivated in minimal media kept at pH 7.2 with 100 mM 3-(*N*-Morpholino)propanesulfonic acid (MOPS) buffer as described before [9,31]. Media contained 24.4 mM methanol. Thiosulfate was added as needed. Antibiotics for *E*. *coli* and *H*. *denitrificans* were used at the following concentrations (in μg ml^−1^): ampicillin, 100; kanamycin, 50; streptomycin, 200; chloramphenicol, 25.

### Recombinant DNA techniques

Standard techniques for DNA manipulation and cloning were used unless otherwise indicated [52]. Restriction enzymes, T4 ligase and Q5 polymerase were obtained from New England Biolabs (Ipswich, United Kingdom) and used according to the manufacturer’s instructions. Oligonucleotides for cloning were obtained from Eurofins MWG (Ebersberg, Germany). Plasmid DNA from *E*. *coli* was purified using the GenJet Plasmid Miniprep kit (Thermo Scientific, Waltham, United States of America). Chromosomal DNA from *H*. *denitrificans* strains was prepared using the Simplex easy DNA kit (GEN-IAL GmbH, Troisdorf, Germany).

### Construction of helper plasmids with lipoate assembly genes from *Thioalkalivibrio* sp. K90mix

For the construction of pACYC-*Tk*lpm (lipoate protein maturation), the potential lipoate assembly genes *lipS1-slpl(AB)-lipT-S2-Y* (TK90_0641–0644, Fig 1C) were amplified with primers TK90_0641–0644 fw and TK90_0641–0644 rev using genomic *Thioalkalivibrio* sp. K90mix DNA as the template. The amplicon was digested with DraI and BspHI and cloned into the EcoRV/BspHI sites of pACYC184. This plasmid contained the *lipS1-slpl(AB)-lipT-lipS2* sequence in the same orientation as the *tet* gene of the vector, thus allowing outreading transcription from the *tet* promoter.

### Production of LbpA proteins in *E*. *coli* BL21 (DE3) Δ*iscR* with and without helper plasmids

Recombinant LbpA proteins were produced in the *E*. *coli* BL21 (DE3) Δ*iscR*, and 500-ml batches of LB medium containing 100 mM MOPS buffer, pH 7.4, 25 mM glucose, and 2 mM iron ammonium citrate as well as ampicillin and kanamycin (and chloramphenicol for strains containing plasmid pACYC-*Tk*lpm) were inoculated with 1% (v/v) *E*. *coli* precultures and cultivated in 1-L flasks at 37°C and 200 rpm until an OD_600_ of 0.8 was reached. At this point, cysteine (0.5 mM), sodium fumarate (25 mM), and IPTG (0.1 mM) were added. Incubation continued for 14 to 16 h at 30°C and 180 rpm. Cells were harvested by centrifugation (11,000 × g, 12 min, 4°C) and lysed by sonication. After removal of insoluble cell material by centrifugation (16,100 × g, 30 min, 4°C), the LbpA proteins were purified by Strep-Tactin affinity according to the manufacturer’s instructions (IBA Lifesciences, Göttingen, Germany). The proteins were then transferred to salt-free 100 mM Tris-HCl (pH 8.0) buffer and underwent further analysis.

### Construction of *H*. *denitrificans* mutant strains

The *tsdA* gene was deleted from *H*. *denitrificans* Δ*lbpA* [2] by using plasmid pK18*mobsacB* ΔtsdATc, transferring it by electroporation, and selecting double crossover recombinants as described previously [9]. For chromosomal integration of the gene encoding HdLbpA2 with a carboxy-terminal His-tag, the modified gene and upstream as well as downstream sequences were amplified by SOE PCR using primers Fwd5′_ΔlbpA, KI_HdLbpA2-His-Up-rev, KI_HdLbpA2-His-Down-fw, and Fwd3′_ΔlbpA (S6 Table). The final plasmid pk18*mobsacB-lbpA2-his*-Tc was transferred into *H*. *denitrificans* Δ*tsdA* Δ*lbpA*. For markerless *in frame* deletion of the *H*. *denitrificans slpl(AB)* gene by splicing overlap extension [53], PCR fragments were constructed using the primers listed in S6 Table. It should be noted that the GTG start codon of the *slpl(AB)* gene overlaps the stop codon of the preceding gene *lipS2* in the sequence GTGA. Similarly, the TGA stop codon of *slpl(AB)* overlaps the start of gene *lipX* in the sequence ATGA. The ribosomal binding site for *lipX* translation must thus be embedded in the *slpl(AB)* gene. To avoid affecting signals for *lipX* translation, the *in frame* deletion of *slpl(AB)* was designed to leave the last 35 bp of the gene untouched. The 2,070 bp fragment, which implements deletion of a 1,029 bp fragment encoding amino acids 8 to 349 of sLpl(AB), was digested with XbaI and cloned into the XbaI site of pk18*mobsacB*-Tc [31]. The final plasmid pk18*mobsacB*Δslpl(AB)-Tc was transferred into *H*. *denitrificans* Δ*tsdA lbpA2-His*. The genotypes of the *H*. *denitrificans* mutant strains generated in this study were confirmed by PCR.

### Purification of His-tagged *Hd*LbpA2

A total of 50 ml *H*. *denitrificans* precultures were grown in 100-ml Erlenmeyer flasks in methanol-containing medium with 100 mM MOPS (pH 7.4), chloramphenicol and streptomycin up to an OD_600_ of 0.7. The main culture had a volume of 1 l in a 2-L Erlenmeyer flask and was inoculated to an OD_600_ of 0.006 with the preculture. The main culture medium contained 2 mM thiosulfate. Cultures were incubated at 30°C and 150 rpm. Cells were harvested by centrifugation at 10,100 × g for 20 min at 4°C, when the cultures were actively oxidizing thiosulfate. Cells were stored at −20°C. Cells were resuspended in 10 ml phosphate buffer (50 mM NaH_2_PO_4_, 300 mM NaCl (pH 7.4)) per g cell material. A spatula tip of DNAse RNAse, lysozyme, and protease inhibitor were added. Cells were broken by ultrasonication at 4°C for 10 min per g cells (Branson sonifier, 55% power) followed by centrifugation (16,100 × g, 4°C, 30 min) and ultracentrifugation (1 h, 4°C, 145,000 × g). Affinity chromatography on 1 ml HisTrap TALON crude columns (Cytiva, Marlborough, Massachusetts, USA) was performed with an Äkta-FPLC system according to the manufacturer’s instructions. Enriched fractions were concentrated with Amicon Ultra-0.5, Ultracel 3k membrane, 3 kDa centrifugal filter units and subjected to electrophoreses on 12.5% Tricine-SDS or on 15% native gels stained with RotiBlue Quick (Carl Roth GmbH, Karlsruhe, Germany) for 1 h. Western blot analysis was performed using nitrocellulose membranes (Amersham Protran 0.2 μm NC, GE Healthcare, Solingen, Germany) and a Trans-Blot Turbo Transfer system (Bio-Rad Laboratories, Munich, Germany). Before western blotting, native gels were incubated for 20 min in Bjerrum buffer (48 mM Tris, 39 mM glycine, 0.04% SDS (pH 9.2)). Whatman filter paper needed during the blotting process was also soaked in Bjerrum buffer. For western blotting of SDS gels, gels and membrane were incubated for 20 min in Towbin buffer (25 mM Tris, 192 mM glycine, 20% (v/v) methanol (pH 8.3)) before loading the blotting chamber. After overnight blocking at 4°C with 4 mM KH_2_PO_4_, 16 mM Na_2_HPO_4_, 115 mM NaCl, pH 7.4 + 0.05% (v/v) Tween 20, 4% milk powder, the membrane was washed 3 times for 5 min in PBS-Tween-buffer (4 mM KH_2_O_4_, 16 mM Na_2_HPO_4_, 115 mM NaCl + 0.1% Tween 20). Proteins were detected with Anti-His-HRP-conjugate (1:5,000) using the SignalFireTM ECL reagent system (Cell Signaling Technology, Cambridge, UK) and visualized with a ChemiDoc Imaging System (BioRad Laboratories, Munich, Germany).

### Characterization of phenotypes, quantification of sulfur compounds, and protein content

Growth experiments with *H*. *denitrificans* were run in in Erlenmeyer flasks with media containing 24.4 mM methanol and varying concentrations of thiosulfate as necessary [31]. Thiosulfate and sulfite concentrations and biomass content were determined by previously described methods [31,54]. All growth experiments were repeated 3 to 5 times. Representative experiments with 2 biological replicates for each strain are shown. All quantifications are based on at least 3 technical replicates.

### Mass spectrometry and Edman degradation

MALDI-TOF measurements and Edman degradation were performed at the Core Facility Protein Synthesis & BioAnalytics, Pharmaceutical Institute, University of Bonn.

### Dataset generation

Archaeal and bacterial genomes were downloaded from GTDB (release R207). In GTDB, all genomes are sorted according to validly published taxonomies, they are pre-validated and have high quality (completeness minus 5*contamination must be higher than 50%). One representative of each of the current 65,703 species clusters was analyzed. It should be noted that GTDB is built on recently standardized bacterial and archaeal taxonomies derived by normalization of the evolutionary distance between taxonomic levels [49,55]. Among the bacteria, 148 phyla are currently distinguished. For the archaea, GTDB lists 16 phyla. Recent reclassification of the archaea was accompanied by merging parts of the Euryarchaeota with the TACK superphylum into a single phylum [49]. Due to these massive but necessary taxonomic rearrangements, conclusions drawn on the distribution and occurrence of genes or groups of genes in higher taxonomic ranks need special care when comparing with previous work. Open reading frames were determined using Prodigal [56] and subsequently annotated for sulfur-related proteins via HMS-S-S [33]. Lipoic acid synthesis proteins and known lipoic acid-dependent enzymes were searched and annotated with HMMs from the TIGRFAMs and Pfams databases with trusted cutoffs. The HMM with the highest above-threshold bitscore was selected for each protein. Lipoate:protein ligases were checked for accessory domain LplB using the pfam PF10437 and annotated accordingly as LplAB. The publicly available HMMs for lipoate synthases, octanoyltransferases, and lipoate:protein ligases are generally sufficiently sensitive to detect all relevant sequences and also precise enough to make a good distinction between related proteins with different functions. However, there are limitations to these HMMs, as they are based on the state of knowledge at the time they were generated and are not updated on a regular basis. Within the lipoate:protein ligases, it is for example not possible to distinguish between LplAB, Lpl(AB), sLpl(AB) from sulfur oxidizers, Lpl(BA), LplJ or Mhp-LplJ. All existing HMMs have been trained by Lpl(AB) and detect catalytic LplA domains of any type even in the absence of the accessory domain LplB. LplJ and LplA sequences are too similar to be distinguished. Thus, we annotated all detected lipoate:protein ligases as Lpl. The main distinguishing feature between the previously known ligases and sLpl(AB) and sLplAB enzymes is the genomic context and/or the type of lipoate synthase present in the organism. LipA and LipS1/S2 can be distinguished very reliably by HMMs.

### Phylogenetic inference

In order to investigate the evolutionary history of the lipoate:protein ligases and octanyoltransferases of the cofactor transferase family PF03099, sequences were separately retrieved from archaeal and bacterial assemblies. A dataset representing classical Lpl(AB) sequences was assembled from LipA-containing genomes not containing *lipS1* or *lipS2*. A second dataset for lipoate:protein ligases originated from gene clusters containing *lipS1*/*lipS2* and a single copy of the ligase gene. Thus, we ensured the highest possible probability that this dataset specifically included LipS1/S2-coupled ligases. LplA and LplB encoded in syntenic gene clusters were concatenated to match the Lpl(AB) domain structure. *Tc*. *kodakarensis* LplAB presented the only exception to this rule and was included in the analysis because the protein is biochemically characterized although the relevant genes do no form a gene cluster. For the circularly permuted LplBA ligases, the sequences of the 2 domains were separated and rejoined in the LplAB order and noted as cpLpl(AB). Octanyoltransferases LipB and LipM from bacterial genomes encoding LipA were clustered by similarity to limit the analysis to a reasonable number of sequences but at the same time maintain the diversity of the underlying dataset, while all corresponding archaeal sequences could be taken into account. A further dataset for LipM octanoyltransferase sequences originated from gene clusters containing *lipS1*/*lipS2* and a single copy of the octanyoltransferase gene, again in order to guarantee the highest possible probability that this dataset specifically includes LipS1/S2-coupled octanoyltransferases.

In order to exclude paralogous sequences, only proteins from assemblies encoding for 1 copy of each, LipS1 and LipS2, were considered and concatenated.

Sequences were aligned using MAFFT [57]. Ambiguously aligned regions and other sources of bias, such as highly variable characters, were removed using BMGE [58] (entropy threshold = 0.95, minimum length = 1, matrix = BLOSUM30), thereby trimming the alignments to regions suitable for phylogenetic inference. Phylogenetic inference by maximum likelihood is widely used in molecular systematics and involves substitution model parameters, branch lengths, and tree topology. In this work, maximum likelihood phylogenies were inferred using IQ-TREE v1.6.12 [59] implemented on the “bonna” high performance clusters of the University of Bonn. As a first step, the best-fitting model of sequence evolution that led to the available data was selected using ModelFinder, a method that combines amino acid substitution model used in other popular model-selection methods [60]. Branch support was then calculated by 3 different tree topology tests, SH-aLRT (2,000 replicates) [61], aBayes (2,000 replicates) [62], and ultrafast bootstrap (2,000 replicates) [63]. Finally, trees were displayed using iTol [64].

Where phylogeny of concatenated sequences was inferred, alignments were made individually and concatenated before trimming by BMGE. As an exception, LplA and LplB encoded in the same gene cluster were concatenated before the alignments as these domains are commonly found to be fused. Octanoyltransferases LipB and LipM lacking a C-terminal accessory domain were added to the LplAB alignments using MAFFT [57]—add-fragment function with–keeplength.

All trees are available in Newick format with associated iTol records in a compressed/zip file archive as Supporting information (S3 Data).

## Supporting information

S1 Fig Structural superposition of *E*. *coli* GcvH with *H*. *denitrificans* LbpA2. Structural data for the *E*. *coli* protein (colored beige) are available in the RCSB Protein Data Bank (accession 3AB9). The structure of the *H*. *denitrificans* LbpA2 (colored light blue) was generated with Alphafold2.
(PDF)

S2 Fig Comparison of bacterial and archaeal lipoate:protein ligases. (**a**) Sequence alignment of Lpl(AB) from *E*. *coli* (Ecoli), sLpl(AB) from *H*. *denitrificans* (Hden), sLpl(AB) from *Thioalkalivibrio* sp. K90mix (TK90), concatenated LplBA from *Thermoplasma acidophilum* acidophilum (Thaci), and concatenated LplBA from *Tc*. *kodakarensis* (Tkoda). The LplB domain/polypeptide is highlighted by a box. The alignment was produced with T-Coffee. Alignment scores are graded from green (bad) to red (good). The right panel shows structural superposition of the *E*. *coli* Lpl(AB) with *H*. *denitrificans* sLplAB (**b**), *Thioalkalivibrio* sp. K90mix sLpl(AB) (**c**), *Thermoplasma acidophilum* LplAB (**d**), and *Thermococcus kodakarensis* LplAB (**e**). All structures were generated with Alphafold2. The *E*. *coli* structure is shown in green in each case. The LplB domain is boxed in all panels.
(PDF)

S3 Fig Mass spectrometric analyses of 3 different recombinant LbpA proteins. *Tk90*LbpA2 from *Thioalkalivibrio* sp. K90mix (**a**), *Ts*LbpA2 (**b**), and *Ts*LbpA1 (**c**) from *Thiorhodospira sibirica* produced in *E*. *coli* BL21 (DE3) Δ*iscR* in the absence (green spectra) or presence (blue spectra) of helper plasmid pACYC-*Tk*lpm carrying genes *lipS1-slpl(AB)-lipT-lipS2* from *Thioalkalivibrio* sp. K90mix. The mass spectrometric data is provided as “S2 Data.”
(PDF)

S4 Fig Phylogeny for clade 3 lipoate:protein ligases including LipM, but excluding cpLpl(BA). Introducing LipM did not change the support of an archaeal origin of clade 3. The data underlying this figure can be found in S3 Data.
(PDF)

S5 Fig Phylogenetic tree of for clade 3 lipoate:protein ligases including cpLpl(BA) but excluding LipM. Introducing cpLpl(BA) did not change the support of an archaeal origin of the clade 3. The data underlying this figure can be found in S3 Data.
(PDF)

S6 Fig Rooted individual phylogenetic trees for LipS2 (a) and LipS1 (b). Sequences were derived from bacterial and archaeal genomes encoding exactly for 1 LipS1 and 1 LipS2. The trees were rooted by biotin synthase BioB as an outgroup. Archaeal sequences are marked in red, bacterial ones in blue. The data underlying this figure can be found in S3 Data.
(PDF)

S1 Table Hidden Markov models for extension of HMS-S-S. (PDF)

S2 Table Presence/absence in bacteria of lipoate synthesis systems, lipoate scavenging, and lipoate requiring proteins. (XLSX)

S3 Table Presence/absence in archaea of lipoate synthesis systems, lipoate scavenging, and lipoate requiring proteins. (XLSX)

S4 Table Numerical data underlying Fig 3C and 3D. (XLSX)

S5 Table List of genomic arrangements. (XLSX)

S6 Table Strains, plasmids, and primers. (PDF)

S1 Data Numerical values underlying Fig 2C and 2D. (XLSX)

S2 Data Mass spectra LbpA proteins. (RAR)

S3 Data Newick treefiles and iTol datasets. (RAR)

S1 Raw Images Raw gel and blot images underlying Fig 2A, 2E and 2F. (PDF)

## Abbreviations

ACP: acyl carrier protein
GTDB: Genome Taxonomy Database
LB: lysogeny broth
SAM: S-adenosylmethionine
