# Supplementary material for: Identification of a novel lipoic acid biosynthesis pathway reveals the complex evolution of lipoate assembly in prokaryotes
Source: PLoS Biol. 2023 Jun 27;21(6):e3002177. doi: 10.1371/journal.pbio.3002177 (PMC10332631; doi:10.1371/journal.pbio.3002177)
Supplement: S1 Fig — Structural data for the E. coli protein (colored beige) are available in the RCSB Protein Data Bank (accession 3AB9). The structure of the H. denitrificans LbpA2 (colored light blue) was generated with Alphafold2. (PDF) [file pbio.3002177.s001.pdf]

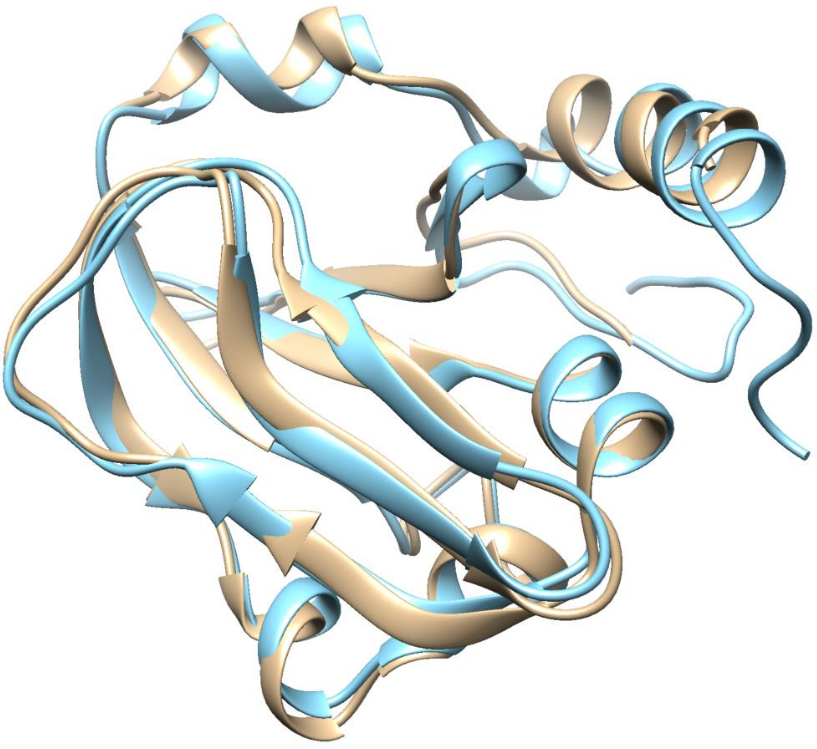

Fig. S1. Structural superposition of *E. coli* GcvH with *H. denitrificans* LbpA2. Structural data for the *E. coli* protein (colored beige) are available in the RCSB Protein Data Bank (accession 3AB9). The structure of the *H. denitrificans* LbpA2 (colored light blue) was generated with Alphafold2.
