# Supplementary material for: Identification of a novel lipoic acid biosynthesis pathway reveals the complex evolution of lipoate assembly in prokaryotes
Source: PLoS Biol. 2023 Jun 27;21(6):e3002177. doi: 10.1371/journal.pbio.3002177 (PMC10332631; doi:10.1371/journal.pbio.3002177)
Supplement: S3 Fig — Tk90LbpA2 from Thioalkalivibrio sp. K90mix (a), TsLbpA2 (b), and TsLbpA1 (c) from Thiorhodospira sibirica produced in E. coli BL21 (DE3) ΔiscR in the absence (green spectra) or presence (blue spectra) of helper plasmid pACYC-Tklpm carrying genes lipS1-slpl(AB)-lipT-lipS2 from Thioalkalivibrio sp. K90mix. The mass spectrometric data is provided as “S2 Data.” (PDF) [file pbio.3002177.s003.pdf]

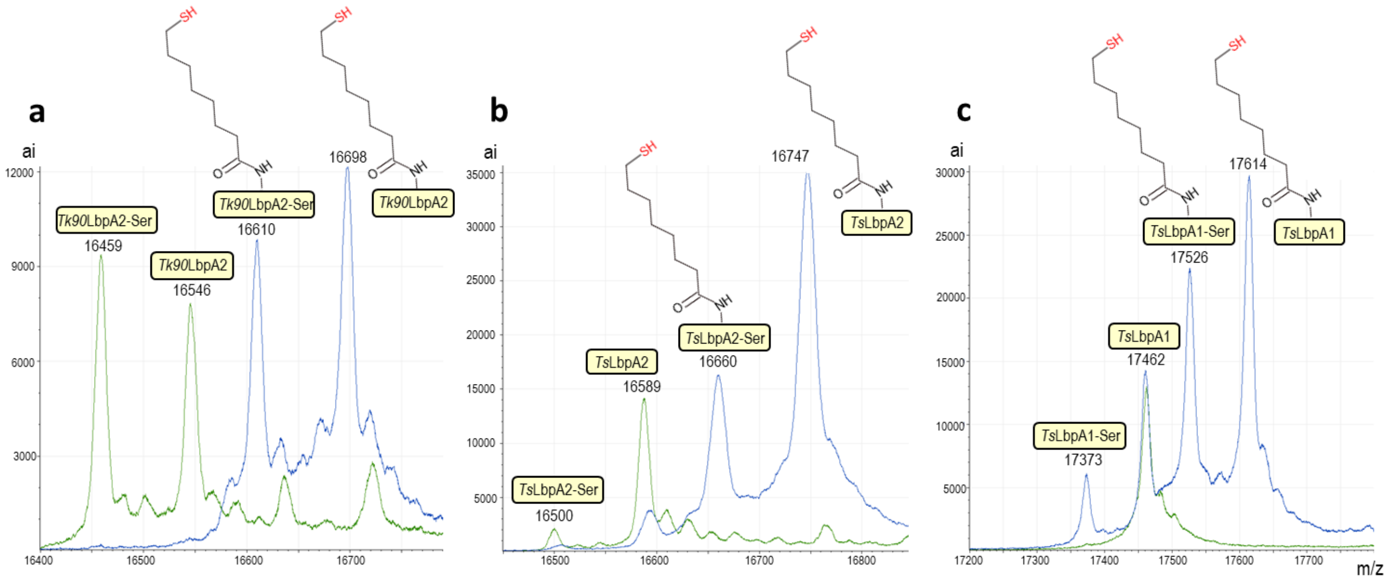

Fig. S3. Mass spectrometric analyses of three different recombinant LbpA proteins (*Tk90*LbpA2 from *Thioalkalivibrio* sp. K90mix (**a**), *Ts*LbpA2 (**b**) and *Ts*LbpA1 (**c**) from *Thiorhodospira sibirica*) produced in *E. coli* BL21 (DE3) Δ*iscR* [1] in the absence (green spectra) or presence (blue spectra) of helper plasmid pACYC-*Tk*lpm carrying genes *lipS1-slpl(AB)-lipT-lipS2-lipY from Thioalkalivibrio* sp. K90mix. The mass spectrometric data is provided as "S2 data.rar". Calculated and obtained masses for the three proteins, their expected modifications and the conditions for detection are given in the table below. The Δ*iscR* strain is engineered for improved synthesis of iron-sulfur proteins like LipS1 and LipS2 by the removal of the gene for IscR, a transcriptional negative regulator of the *isc* (iron-sulfur cluster biosynthesis) operon. Predicted masses for the recombinant apo-proteins were 16547.47 Da (*Tk90*LbpA2), 16589.64 Da (*Ts*LbpA2) and 17465.71 Da (*Ts*LbpA1). In addition to the full length apo-proteins, polypeptides with masses 87-89 Da lower were recovered. This mass difference is consistent with the absence of the amino-terminal amino acid serine, derived from the Strep-tag in part of the protein population. Our hypothesis was confirmed by Edman degradation of *Tk90*LbpA2. In the presence of the helper plasmid, all LbpA protein species analyzed exhibited masses 151-160 Da higher, which is fully compatible with the addition of a monothiolated octanoyl moiety. Based on the results obtained for the archaeal LipS1/S2 proteins [2], we suggest that this is the 8-mercaptooctanyl intermediate.

Protein	Modification	Calculated/obtained mass [Da]	Condition for obtained mass
***TK90*LbpA2**	Apo-protein	16547/16546 (Δ: 1)	Without helper plasmid
	Octanoylated	16672/nd	Not Detected
	Monothiolated octanoyl	16704/16698 (Δ: 6)	With helper plasmid
	Holo-protein	16736/nd	Not Detected
	Apo-protein-Ser	16459/16459 (Δ: 0)	Without helper plasmid
	Octanoylated-Ser	16584/nd	Not Detected
	Monothiolated octanoyl-Ser	16616/16610 (Δ: 6)	With helper plasmid
	Holo-protein-Ser	16648/n	Not Detected
***Ts*LbpA2**	Apo-protein	16589/16589 (Δ: 0)	Without and with helper plasmid
	Octanoylated	16714/nd	Not Detected
	monothiolated octanoyl	16746/16747 (Δ: 1)	With helper plasmid
	Holo-protein	16778/nd	Not detected
	Apo-protein-Ser	16501/16500 (Δ: 1)	Without helper plasmid
	Octanoylated-Ser	16626/nd	Not detected
	Monothiolated octanoyl -Ser	16658/16660 (Δ: 2)	With helper plasmid
	Holo-protein-Ser	16690/nd	Not detected
***Ts*LbpA1**	Apo-protein	17465/17462 (Δ: 3)	Without and with helper plasmid
	Octanoylated	17590/nd	Not detected
	Monothiolated octanoyl	17622/17614 (Δ: 8)	With helper plasmid
	Holo-protein	17654/nd	Not detected
	Apo-protein -Ser	17377/17373 (Δ: 4)	With helper plasmid
	Octanoylated-Ser	17502/nd	Not detected
	Monothiolated octanoyl -Ser	17534/17526 (Δ: 8)	With helper plasmid
	Holo-protein-Ser	17566/nd	Not detected

References

1. Akhtar MK, Jones PR. Deletion of *iscR* Stimulates Recombinant Clostridial Fe-Fe Hydrogenase Activity and H_2-Accumulation in *Escherichia coli* BL21(DE3). *Appl. Microbiol. Biotechnol.* 2008; 78(5):853-862. https://doi.org/10.1007/s00253-008-1377-6
2. Neti SS, Sil D, Warui DM, Esakova OA, Solinski AE, Serrano DA, et al. Characterization of LipS1 and LipS2 from *Thermococcus kodakarensis*: Proteins Annotated as Biotin Synthases, Which Together Catalyze Formation of the Lipoyl Cofactor. *ACS Bio Med Chem Au*. 2022; 2(5):509-520. https://doi.org/10.1021/acsbiomedchemau.2c00018
