# Supplementary material for: Identification of a novel lipoic acid biosynthesis pathway reveals the complex evolution of lipoate assembly in prokaryotes
Source: PLoS Biol. 2023 Jun 27;21(6):e3002177. doi: 10.1371/journal.pbio.3002177 (PMC10332631; doi:10.1371/journal.pbio.3002177)
Supplement: S4 Fig — Introducing LipM did not change the support of an archaeal origin of clade 3. The data underlying this figure can be found in S3 Data. (PDF) [file pbio.3002177.s004.pdf]

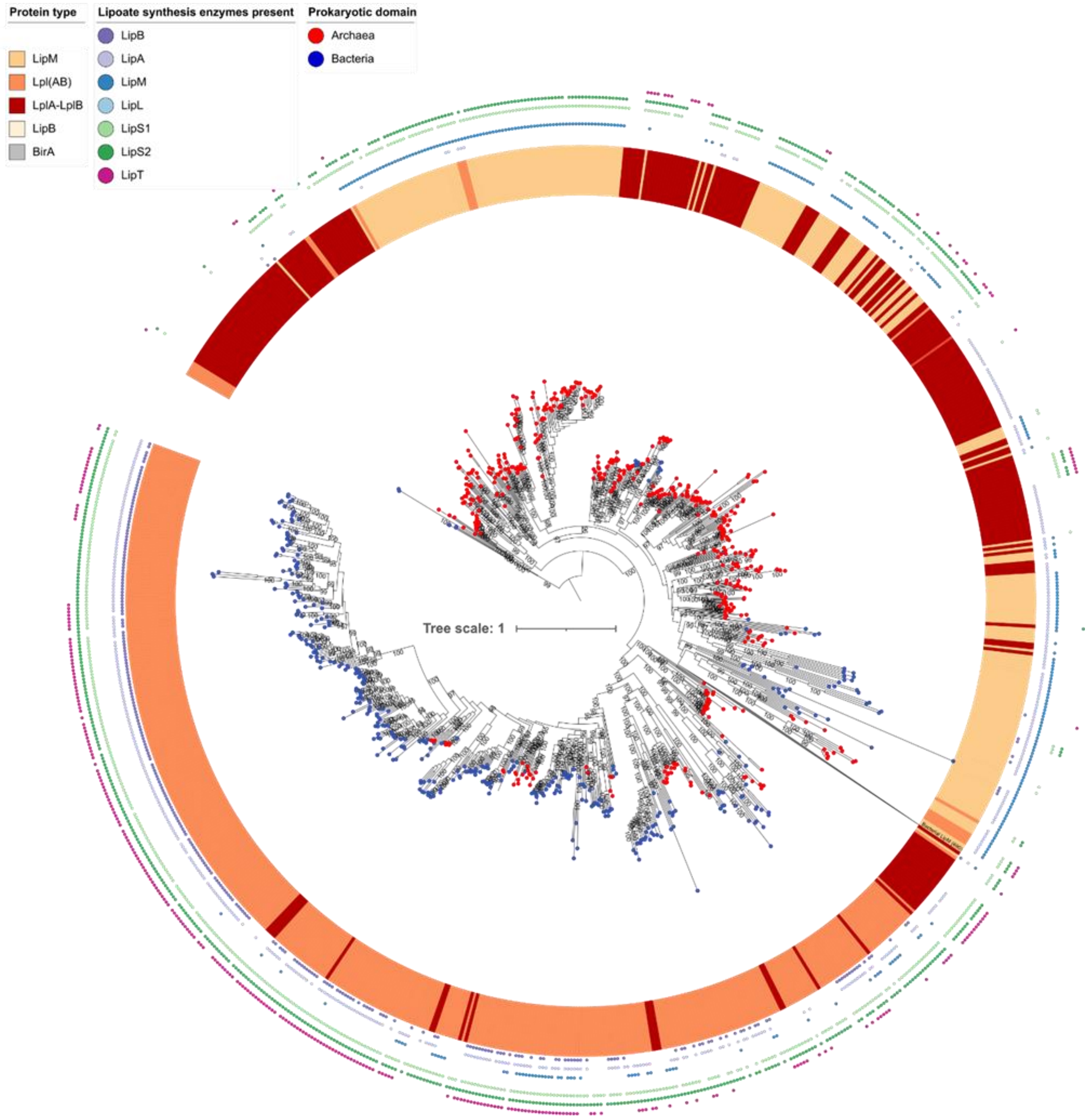

Fig. S4. **Phylogeny for clade 3 lipoate:protein ligases including LipM, but excluding cpLpl(BA).** Introducing LipM did not change the support of an archaeal origin of clade 3. The data underlying this Figure can be found in Supplementary data S3.
