# Supplementary material for: Identification of a novel lipoic acid biosynthesis pathway reveals the complex evolution of lipoate assembly in prokaryotes
Source: PLoS Biol. 2023 Jun 27;21(6):e3002177. doi: 10.1371/journal.pbio.3002177 (PMC10332631; doi:10.1371/journal.pbio.3002177)
Supplement: S5 Fig — Introducing cpLpl(BA) did not change the support of an archaeal origin of the clade 3. The data underlying this figure can be found in S3 Data. (PDF) [file pbio.3002177.s005.pdf]

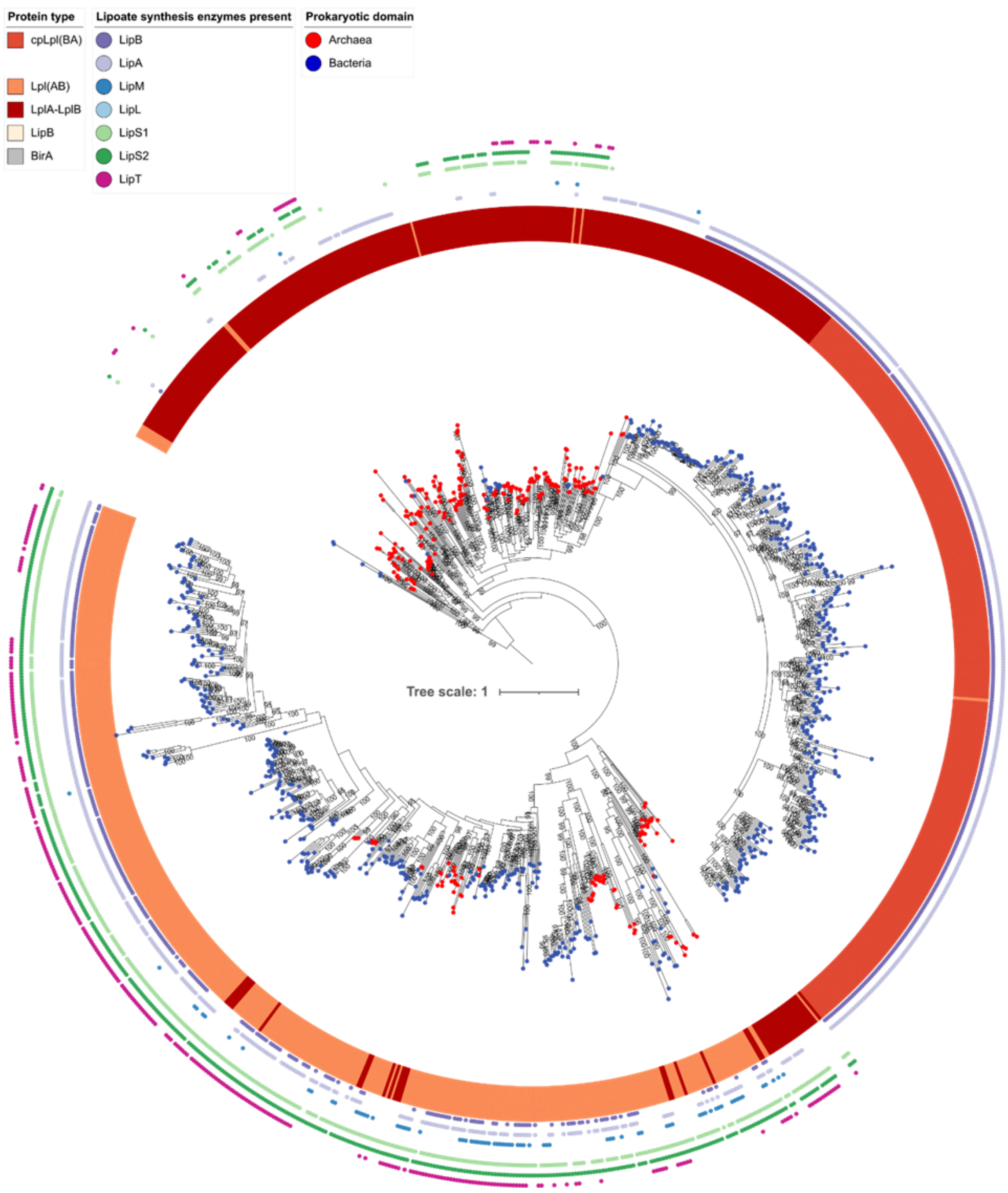

Fig. S5. Phylogenetic tree of for clade 3 lipoate:protein ligases including cpLpl(BA) but excluding LipM. Introducing cpLpl(BA) did not change the support of an archaeal origin of the clade 3. The data underlying this Figure can be found in Supplementary data S3.
