# Supplementary material for: Identification of a novel lipoic acid biosynthesis pathway reveals the complex evolution of lipoate assembly in prokaryotes
Source: PLoS Biol. 2023 Jun 27;21(6):e3002177. doi: 10.1371/journal.pbio.3002177 (PMC10332631; doi:10.1371/journal.pbio.3002177)
Supplement: S6 Fig — Sequences were derived from bacterial and archaeal genomes encoding exactly for 1 LipS1 and 1 LipS2. The trees were rooted by biotin synthase BioB as an outgroup. Archaeal sequences are marked in red, bacterial ones in blue. The data underlying this figure can be found in S3 Data. (PDF) [file pbio.3002177.s006.pdf]

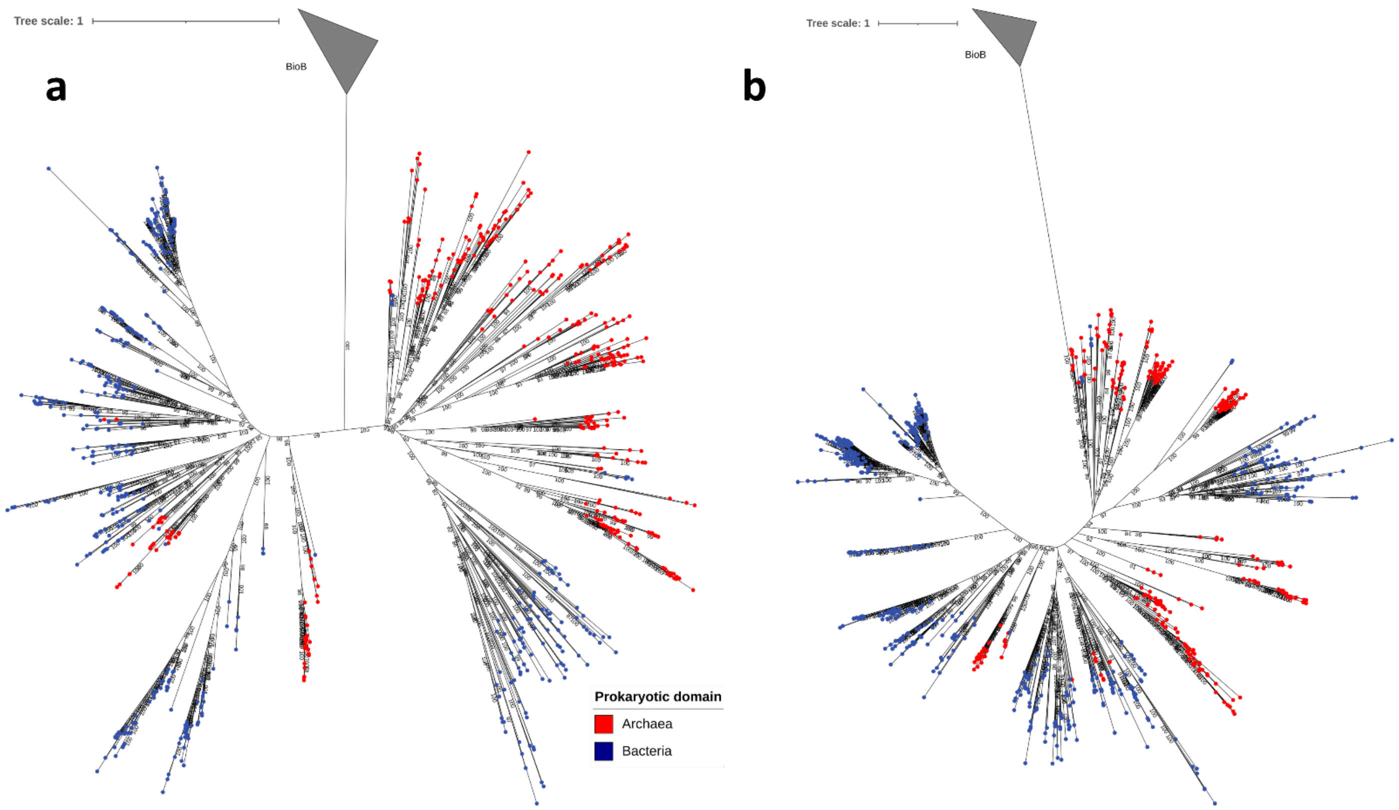

Fig. S6. Rooted individual phylogenetic trees for LipS2 (a) and LipS1 (b). Sequences were derived from bacterial and archaeal genomes encoding exactly for one LipS1 and one LipS2. The trees were rooted by biotin synthase BioB as an outgroup. Archaeal sequences are marked in red, bacterial ones in blue. The data underlying this Figure can be found in Supplementary data S3.
