# Supplementary material for: Identification of a novel lipoic acid biosynthesis pathway reveals the complex evolution of lipoate assembly in prokaryotes
Source: PLoS Biol. 2023 Jun 27;21(6):e3002177. doi: 10.1371/journal.pbio.3002177 (PMC10332631; doi:10.1371/journal.pbio.3002177)
Supplement: S1 Table — (PDF) [file pbio.3002177.s007.pdf]

Table S1. Hidden Markov Models for extension of HMS-S-S [1]

Hidden Markov Model	Threshold score	Source	Enzyme
TIGR00121_birA	83.1	Tigrfam	Bifunctional ligase/repressor BirA
TIGR00214_LipB	83.55	Tigrfam	octanoyltransferase
TIGR00510_LipA	310.25	Tigrfam	lipoate synthase
TIGR00545_LplA	201.85	Tigrfam	lipoate:protein ligase
PRK05481_LipA	203	InterPro	lipoate synthase
PF10437_LplB	24	pfam	accessory domain lipoate:protein ligase
PF03099_Lpl-Lip-bpl	21	pfam	small cofactor transferase family
IPR024897_LipL	100	InterPro	amidotransferase
cd16444_LipB	191.17	InterPro	octanoyltransferase
MF02118_LipM	130	HAMAP	octanoyltransferase
MF02119_LipL	100.1	HAMAP	amidotransferase
cd16443_LplA	100.1	InterPro	lipoate:protein ligase
cd16442_BPL	115	InterPro	biotin:protein ligase
TIGR00461_GcvP	859.2	Tigrfam	glycine cleavage P protein
TIGR00527_GcvH	115.85	Tigrfam	glycine cleavage H protein
TIGR00528_GcvT	235.85	Tigrfam	glycine cleavage T protein
TIGR00759_PdhE1	859.9	Tigrfam	pyruvat dehydrogenase E1
TIGR01348_PdhE2	552.1	Tigrfam	pyruvat dehydrogenase E2
TIGR01350_DHDL	408.8	Tigrfam	Dihydrolipoamide dehydrogenase
TIGR03181_PdhE1a	411.35	Tigrfam	pyruvat dehydrogenase E1
TIGR03182_PdhE1a	393.85	Tigrfam	pyruvat dehydrogenase E1
PRK09405_PdhE1	1326	InterPro	pyruvat dehydrogenase E1
cd02017_TPP_PdhE1	479	InterPro	pyruvat dehydrogenase E1
PRK09212_PdhE1b	321	InterPro	pyruvat dehydrogenase E1
PRK11855_PdhE2	576	InterPro	pyruvat dehydrogenase E2
PRK09404_KdhE1	764	InterPro	a-ketoglutarate dehydrogenase E1
PRK05704_KdhE2	449	InterPro	a-ketoglutarate dehydrogenase E2
TIGR00239_KdhE1	770	Tigrfam	a-ketoglutarate dehydrogenase E1
TIGR03186_KdhE2	1408	Tigrfam	a-ketoglutarate dehydrogenase E2
PF12573_BcdhE1a_N	26	pfam	branched-chain dehydrogenase E1
PRK11856_BcdhE2	250	InterPro	branched-chain dehydrogenase E1
PRK05976_DHDL	469	InterPro	Dihydrolipoamide dehydrogenase
PRK06327_DHDL	639	InterPro	Dihydrolipoamide dehydrogenase
PRK06467_DHDL	752	InterPro	Dihydrolipoamide dehydrogenase
PRK06912_DHDL	374	InterPro	Dihydrolipoamide dehydrogenase
PRK07818_DHDL	389	InterPro	Dihydrolipoamide dehydrogenase
PRK07846_DHDL	501	InterPro	Dihydrolipoamide dehydrogenase
COG1071_AodhE1	283	eggNOG	acetoin dehydrogenase
COG0022_AodhE1b	406	eggNOG	acetoin dehydrogenase
PRK14875_AodhE2	346.9	InterPro	acetoin dehydrogenase

References

1. Tanabe TS, Dahl C. HMS-S-S: A Tool for the Identification of Sulphur Metabolism-Related Genes and Analysis of Operon Structures in Genome and Metagenome Assemblies. *Mol. Ecol. Resour.* 2022; 22(7):2758-2774. https://doi.org/10.1111/1755-0998.13642
