# Supplementary material for: Identification of a novel lipoic acid biosynthesis pathway reveals the complex evolution of lipoate assembly in prokaryotes
Source: PLoS Biol. 2023 Jun 27;21(6):e3002177. doi: 10.1371/journal.pbio.3002177 (PMC10332631; doi:10.1371/journal.pbio.3002177)
Supplement: S6 Table — (PDF) [file pbio.3002177.s012.pdf]

Table S6. Strains, plasmids and primers. The locus tags given for *Ts. sibirica* refer to IMG JGI nomenclature.

Strains primers or plasmids	Relevant genotype, description or sequence	Reference or source
**Strains**		
Thiorhodospira sibirica ATCC 700588^T	Wild type	[1]
Thioalkalivibrio sp. K90 mix	Wild type	[2]
Hyphomicrobium denitrificans ATCC 51888^T	Wild type	[3]
E. coli 10β	*Δ(ara-leu) 7697 araD139 fhuA ΔlacX74 galK16 galE15 e14- φ80dlacZΔM15 recA1 relA1 endA1 nupG rpsL* (Str^R) *rph spoT1 Δ(mrr-hsdRMS-mcrBC)*	New England Biolabs
E. coli BL21 (DE3)	*F$^-$ ompT hsdS$_B$(r$_B$$^-m_B$$^-$) gal dcm* (DE3)	Novagen
E. coli BL21 (DE3) Δ*iscR*	F$^-$ *ompT hsdS*$_B$(*r$_B$$^-m_B$$^-$*) *gal dcm iscrR*::kan (DE3)	[4]
H. denitrificans Δ*tsdA*	In-frame deletion of *tsdA* in *H. denitrificans* ATCC 51888	[5]
H. denitrificans Δ*tsdA* Δ*lbpA*	In-frame deletion of *lpbA* (Hden_0696) in *H. denitrificans* Δ*tsdA*	This work
H. denitrificans Δ*tsdA lbpA-His*	Complementation of *lbpA-His* into strain *Hyphomicrobium denitrificans* Δ*tsdA* Δ*lbpA*	This work
H. denitrificans Δ*tsdA* Δ*slpl(AB) lbpA-His*	In-frame deletion of *slplA* (Hden_686) in in *H. denitrificans* Δ*tsdA lbpA-His*	This work
**Primers**		
THISIDRAFT_1817 (*lbpA2*) fw	TTTT**CATATG**AGCGCTTGGAGCCACCCGCAGTTCGAA AAAGGCGCCGGCACTGCAAAAGGC (NdeI)	This work
THISIDRAFT_1817 (*lbpA2*) rev	TTTT**CTCGAG**TTAAGCCCCGCACCC (XhoI)	This work
TK90_0638 (*lbpA1*) strep_fw	TTTT**CATATG**AGCGCTTGGAGCCACCCGCAGTTCGAA AAAGGCGCCGACTGCAACGGTTGC (NdeI)	This work
TK90_0638 (*lbpA1*) strep_rev	TTTT**CTCGAG**CTATCGCAGCTTGCGCTG (XhoI)	This work
TK90_0640 (*lbpA2*) strep_fw	TTTT**CATATG**AGCGCTTGGAGCCACCCGCAGTTCGAA AAAGGCGCCGGCGCAGTACGGGGT (NdeI)	This work
TK90_0640 (*lbpA2*) strep_rev	TTTT**CTCGAG**TTAGCAGCCGCCGAA (XhoI)	This work
TK90_0641-0644 fw	GCAG**TTTAAA**CCGGAGCAAGCTGATGAC (DraI)	This work
TK90_0641-0644 rev	GCA**TCATGA**TTATGCGGACTTCTCGTCC (BspHI)	This work
Hden_0686_fw-up	ATAT**TCTAGA**ATCTGCTGCTGAC ATATCCTGAAGG (XbaI)	This work
Hden_0686_fw-down	GTGAGTATAGGGCGGCGCGCAACCGTGGTTCACGCG GCTTT	This work
Hden_0686_rev-up	AAAGCCGCGTGAACCACGGTTGCGCGCCGCCCTATAC TCAC	This work
Hden_0686_rev-down	ATAT**TCTAGA**GTGCAAATGACCTCGACGTCGTAGC (XbaI)	This work
Fwd5'_ΔlbpA	AAAA**GCATGC**CACCAAGGGACGGCTCGCT (SphI)	[6]
Fwd3'_ΔlbpA	AAAA**TCTAGA**GCGATCGCTCGATGGAAAA (XbaI)	[6]
KI_HdLbpA2-His-Up-rev	TCAGTGGTGGTGGTGGTGGTGGCCGCCGCCGCCGCC GCAACCTGCGAAAC	Tanabe
KI_HdLbpA2-His-Down-fw	GGCGGCGGCGGCCACCACCACCACCACCACTGAGCT CTACGGCCGCTCT	Tanabe
**Plasmids**		
pET22b	Ap^r	Novagen
pACYC184	Cm^r, Tc^r	[7]
pACYC-*Tk*lpm	Cm^r, DraI/BspHI fragment covering *lipS1-slpl(AB)-lipT-lipS2* from *Thioalkalivibrio* sp. K90mix in EcoRV/BspHI of pACYC184	This work
pET15b	Ap^r	Novagen
pET22b	Ap^r	Novagen
pET28b	Km^r	Novagen

pHP45Ω-Tc	Ap^r, Tc^r	[8]
pk18*mobsacB*-Tc	pHP45ΩTc tetracycline cassette inserted into pk18*mobsacB* using SmaI	[9]
pk18*mobsacB*ΔtsdATc	Km^r, Tc^r, 2.01 kb fragment implementing deletion of a 996 bp *tsdA* fragment in pK18*mobsacB* with additional tetracycline resistance	[5]
pk18*mobsacB*ΔslplA-Tc	Km^r, Tc^r, 2.07 kb SOE PCR fragment implementing deletion of a 1029 bp fragment encoding amino acids 8 to 349 of sLplpA cloned into pk18mobsacB-Tc using XbaI restriction sites	This work
pk18*mobsacB-lbpA-His*	Km^r, SOE PCR fragment implementing chromosomal integration of *lbpA* joined with a His-tag encoding sequence cloned into pk18mobsacB using XbaI and SphI restriction sites	This work
pk18*mobsacB-lbpA-His*-Tc	Km^r, Tc^r, pk18*mobsacB-lbpA-his* with tetracycline resistance gene from pHP45Ω cloned into SmaI site	This work
pET-*Ts*LbpA1-N-Strep	Ap^r, N-terminal Strep-tag, NdeI-EcoRI fragment of PCR amplified *lbpA1* from *Thiorhodospira sibirica* (THISIDRAFT_ RS04590, former ThisiDRAFT_1533) in pET22b	[6]
pET-*Ts*LbpA2-N-Strep	Ap^r, N-terminal Strep-tag, NdeI-XhoI fragment of PCR amplified *lbpA2* from *Thiorhodospira sibirica* (THISIDRAFT_ RS08775) in pET22b	This work
pET-*TK90*LbpA2-N-Strep	Ap^r, N-terminal Strep-tag, NdeI-XhoI fragment of PCR amplified *lbpA2* from *Thioalkalivibrio* sp. K90mix (TK90_0640) in pET22b	This work

References

1. Bryantseva IA, Gorlenko VM, Kompantseva EI, Imhoff JF, Süling J, Mityushina L. *Thiorhodospira sibirica* gen. nov., sp. nov., a New Alkaliphilic Purple Sulfur Bacterium from a Siberian Soda Lake. *Int. J. Syst. Bacteriol.* 1999; 49:697-703. https://doi.org/10.1099/00207713-49-2-697
2. Muyzer G, Sorokin DY, Mavromatis K, Lapidus A, Foster B, Sun H, et al. Complete Genome Sequence of *Thioalkalivibrio* sp. K90mix. *Stand. Genomic Sci.* 2011; 5(3):doi:10.4056/sigs.2315092. https://doi.org/10.4056/sigs.2315092
3. Urakami T, Sasaki J, Suzuki KI, Komagata K. Characterization and Description of *Hyphomicrobium denitrificans* sp. nov. *Int. J. Syst. Bacteriol.* 1995; 45(3):528-532. https://doi.org/10.1099/00207713-45-3-528
4. Akhtar MK, Jones PR. Deletion of *iscR* Stimulates Recombinant Clostridial Fe-Fe Hydrogenase Activity and H_2-Accumulation in *Escherichia coli* BL21(DE3). *Appl. Microbiol. Biotechnol.* 2008; 78(5):853-862. https://doi.org/10.1007/s00253-008-1377-6
5. Koch T, Dahl C. A Novel Bacterial Sulfur Oxidation Pathway Provides a New Link between the Cycles of Organic and Inorganic Sulfur Compounds. *ISME J.* 2018; 12(10):2479-2491. https://doi.org/10.1038/s41396-018-0209-7
6. Cao X, Koch T, Steffens L, Finkensieper J, Zigann R, Cronan JE, et al. Lipoate-Binding Proteins and Specific Lipoate-Protein Ligases in Microbial Sulfur Oxidation Reveal an Atpyical Role for an Old Cofactor. *eLife*. 2018; 7:e37439. https://doi.org/10.7554/eLife.37439
7. Chang AC, Cohen SN. Construction and Characterization of Amplifiable Multicopy DNA Cloning Vehicles Derived from the p15a Cryptic Miniplasmid. *J. Bacteriol.* 1978; 134(3):1141-1156.
8. Fellay R, Frey J, Krisch HM. Interposon Mutagenesis of Soil and Water Bacteria: A Family of DNA Fragments Designed for in Vitro Insertional Mutagenesis of Gram-Negative Bacteria. *Gene*. 1987; 52:147-154. https://doi.org/10.1016/0378-1119(87)90041-2
9. Li J, Koch J, Flegler W, Garcia Ruiz L, Hager N, Ballas A, et al. A Metabolic Puzzle: Consumption of C_1 Compounds and Thiosulfate in *Hyphomicrobium denitrificans* X^t. *Biochim. Biophys. Acta - Bioenergetics*. 2023; 1864:148932. https://doi.org/10.1016/j.bbabio.2022.148932
