# Supplementary material for: Identification of a novel lipoic acid biosynthesis pathway reveals the complex evolution of lipoate assembly in prokaryotes
Source: PLoS Biol. 2023 Jun 27;21(6):e3002177. doi: 10.1371/journal.pbio.3002177 (PMC10332631; doi:10.1371/journal.pbio.3002177)

Figure 2a, lanes on the left

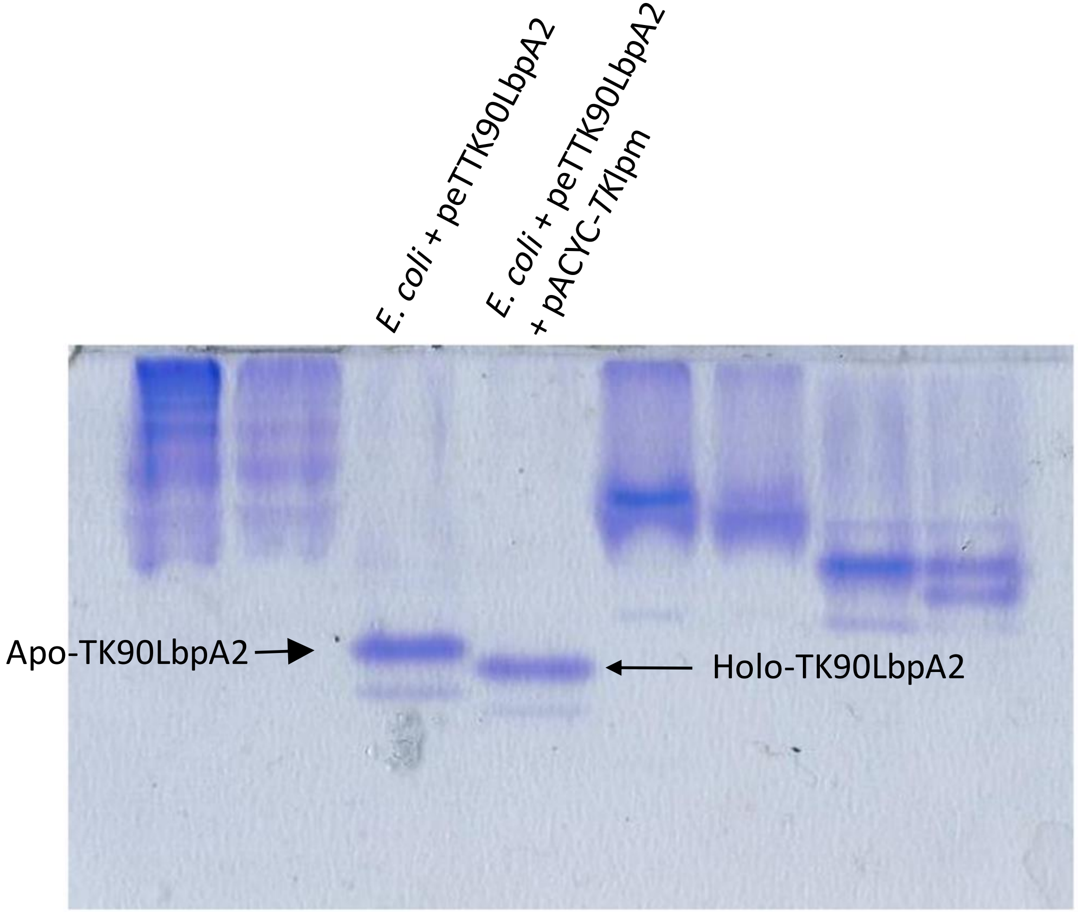

Figure 2a, lanes on right

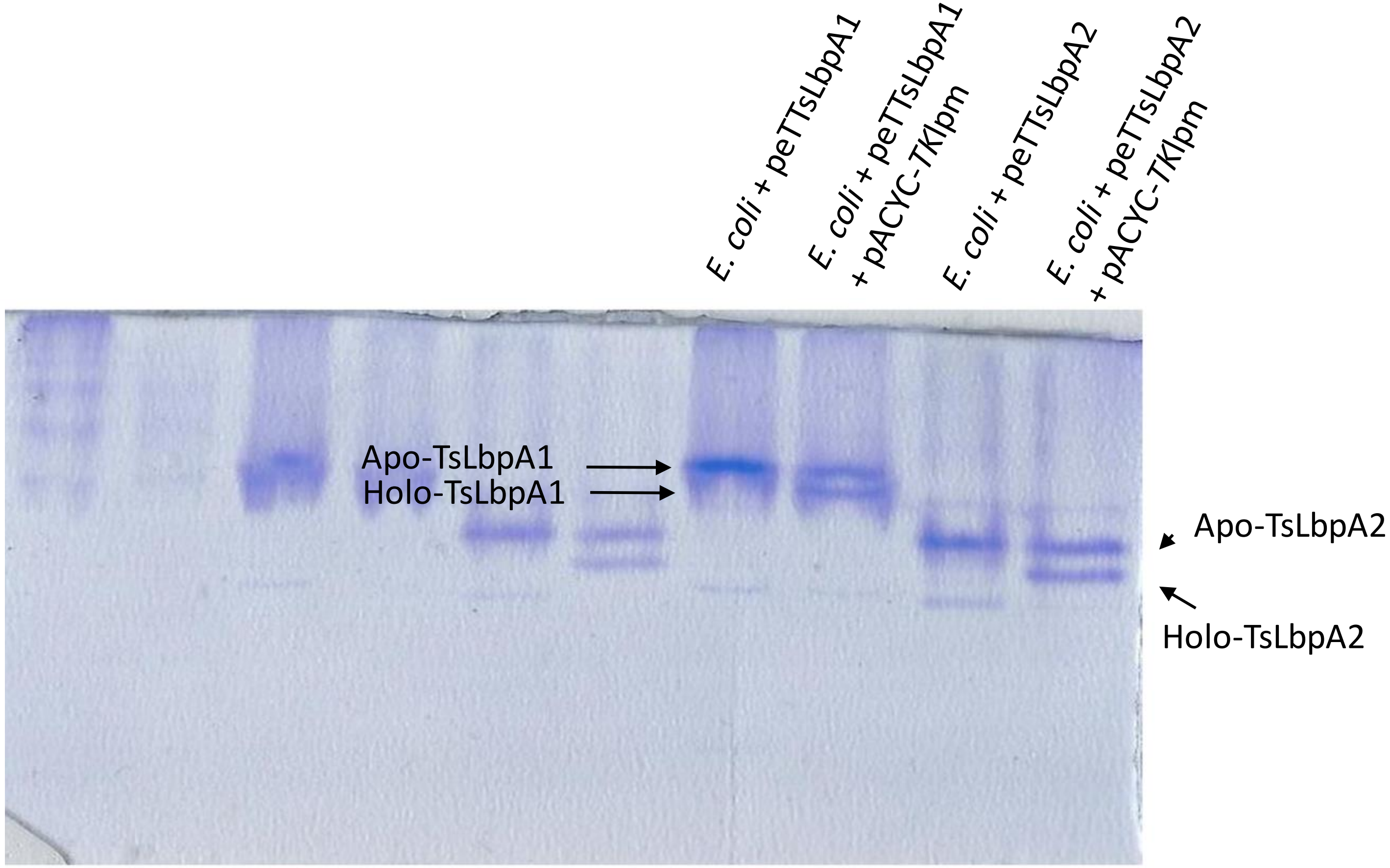

Figure 2e

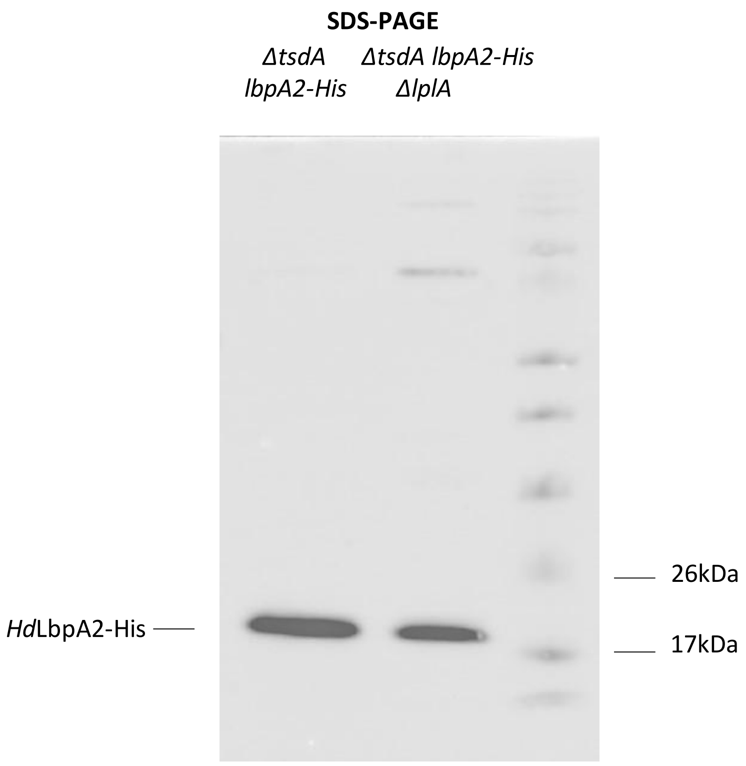

Figure 2f

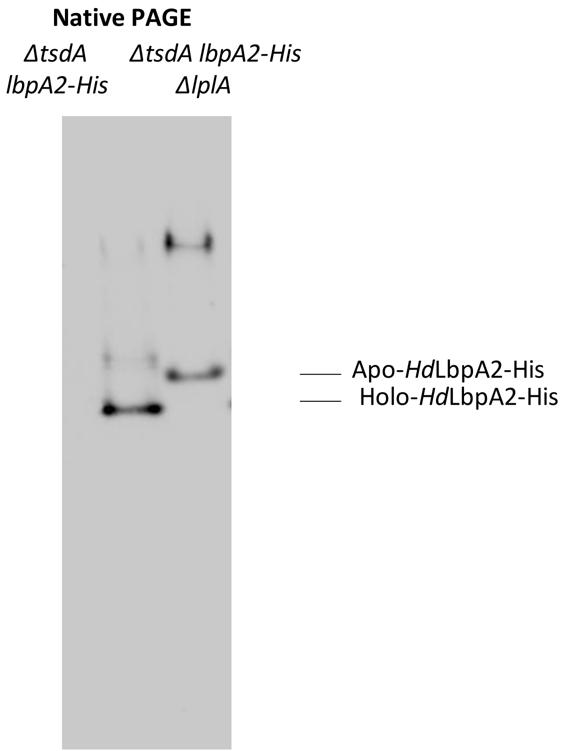

Supplement: S1 Raw Images — (PDF) [file pbio.3002177.s016.pdf]
