# Supplementary material for: Identification of a novel lipoic acid biosynthesis pathway reveals the complex evolution of lipoate assembly in prokaryotes
Source: PLoS Biol. 2023 Jun 27;21(6):e3002177. doi: 10.1371/journal.pbio.3002177 (PMC10332631; doi:10.1371/journal.pbio.3002177)
Supplement: S2 Fig — (a) Sequence alignment of Lpl(AB) from E. coli (Ecoli), sLpl(AB) from H. denitrificans (Hden), sLpl(AB) from Thioalkalivibrio sp. K90mix (TK90), concatenated LplBA from Thermoplasma acidophilum acidophilum (Thaci), and concatenated LplBA from Tc. kodakarensis (Tkoda). The LplB domain/polypeptide is highlighted by a box. The alignment was produced with T-Coffee. Alignment scores are graded from green (bad) to red (good). The right panel shows structural superposition of the E. coli Lpl(AB) with H. denitrificans sLplAB (b), Thioalkalivibrio sp. K90mix sLpl(AB) (c), Thermoplasma acidophilum LplAB (d), and Thermococcus kodakarensis LplAB (e). All structures were generated with Alphafold2. The E. coli structure is shown in green in each case. The LplB domain is boxed in all panels. (PDF) [file pbio.3002177.s002.pdf]

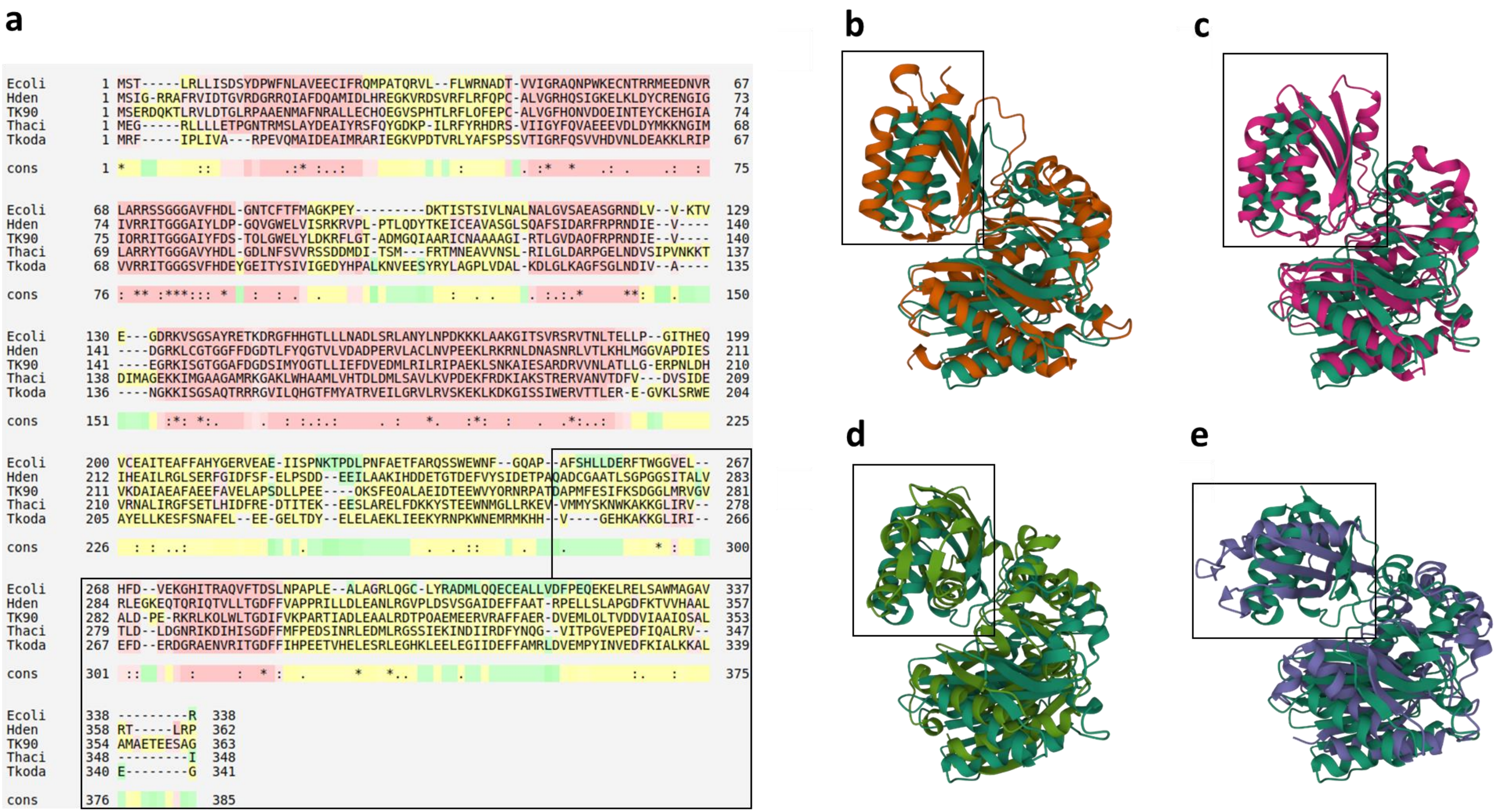

Fig S2. Comparison of bacterial and archaeal lipoate:protein ligases. (**a**) Sequence alignment of Lpl(AB) from *E. coli* (Ecoli), sLpl(AB) from *H. denitrificans* (Hden), sLpl(AB) from *Thioalkalivibrio* sp. K90mix (TK90), concatenated LplBA from *Tp. acidophilum* (Thaci), and concatenated LplBA from *Tc. kodakarensis* (Tkoda). The LplB domain/polypeptide is highlighted by a box. The alignment was produced with T-Coffee. Alignment scores are graded from green (bad) to red (good). The right panel shows structural superposition of the *E. coli* Lpl(AB) with *H. denitrificans* sLplAB (**b**), *Thioalkalivibrio* sp. K90mix sLpl(AB) (**c**) *Thermoplasma acidophilum* LplAB (**d**) and *Thermococcus kodakarensis* LplAB (**e**). All structures were generated with Alphafold2. The *E. coli* structure is shown in green in each case. The LplB domain is boxed in all panels.
